# Age and sex-associated variation in the multi-site microbiome of an entire social group of free-ranging rhesus macaques

**DOI:** 10.1186/s40168-021-01009-w

**Published:** 2021-03-22

**Authors:** Mareike C. Janiak, Michael J. Montague, Catalina I. Villamil, Michala K. Stock, Amber E. Trujillo, Allegra N. DePasquale, Joseph D. Orkin, Samuel E. Bauman Surratt, Olga Gonzalez, Michael L. Platt, Melween I. Martínez, Susan C. Antón, Maria Gloria Dominguez-Bello, Amanda D. Melin, James P. Higham

**Affiliations:** 1 Department of Anthropology and Archaeology, University of Calgary, Alberta, Canada; 2 Alberta Children’s Hospital Research Institute, Alberta, Canada; 3 Department of Anthropology, New York University, New York, USA; 4 School of Science, Engineering and Environment, University of Salford, Salford, UK; 5 Department of Neuroscience, University of Pennsylvania, Philadelphia, PA USA; 6 School of Chiropractic, Universidad Central del Caribe, Bayamón, Puerto Rico; 7 Department of Sociology and Anthropology, Metropolitan State University of Denver, Denver, CO USA; 8 New York Consortium in Evolutionary Primatology, New York, NY USA; 9 Institut de Biologia Evolutiva, Universitat Pompeu Fabra-CSIC, Barcelona, Spain; 10 Caribbean Primate Research Center, University of Puerto Rico, San Juan, Puerto Rico; 11 Disease Intervention and Prevention, Southwest National Primate Research Center, San Antonio, TX USA; 12 Department of Biochemistry and Microbiology, Rutgers University, New Brunswick, NJ USA; 13 Department of Anthropology, Rutgers University, New Brunswick, NJ USA; 14 Department of Medical Genetics, University of Calgary, Alberta, Canada

**Keywords:** Aging, Non-human primates, Genital microbiome, Oral microbiome, Gut microbiome, Sex differences

## Abstract

**Background:**

An individual’s microbiome changes over the course of its lifetime, especially during infancy, and again in old age. Confounding factors such as diet and healthcare make it difficult to disentangle the interactions between age, health, and microbial changes in humans. Animal models present an excellent opportunity to study age- and sex-linked variation in the microbiome, but captivity is known to influence animal microbial abundance and composition, while studies of free-ranging animals are typically limited to studies of the fecal microbiome using samples collected non-invasively. Here, we analyze a large dataset of oral, rectal, and genital swabs collected from 105 free-ranging rhesus macaques (*Macaca mulatta*, aged 1 month-26 years), comprising one entire social group, from the island of Cayo Santiago, Puerto Rico. We sequenced 16S V4 rRNA amplicons for all samples.

**Results:**

Infant gut microbial communities had significantly higher relative abundances of *Bifidobacterium* and *Bacteroides* and lower abundances of *Ruminococcus*, *Fibrobacter*, and *Treponema* compared to older age groups, consistent with a diet high in milk rather than solid foods. The genital microbiome varied widely between males and females in beta-diversity, taxonomic composition, and predicted functional profiles. Interestingly, only penile, but not vaginal, microbiomes exhibited distinct age-related changes in microbial beta-diversity, taxonomic composition, and predicted functions. Oral microbiome composition was associated with age, and was most distinctive between infants and other age classes.

**Conclusions:**

Across all three body regions, with notable exceptions in the penile microbiome, while infants were distinctly different from other age groups, microbiomes of adults were relatively invariant, even in advanced age. While vaginal microbiomes were exceptionally stable, penile microbiomes were quite variable, especially at the onset of reproductive age. Relative invariance among adults, including elderly individuals, is contrary to findings in humans and mice. We discuss potential explanations for this observation, including that age-related microbiome variation seen in humans may be related to changes in diet and lifestyle.

Video abstract

**Supplementary Information:**

The online version contains supplementary material available at 10.1186/s40168-021-01009-w.

## Introduction

A major goal of the biomedical sciences is to understand how life transitions and aging impact human biological processes, health, and wellness. In the past decade, a growing body of literature has focused on the key role that microbial communities play in these processes, in the hopes of identifying targets for medical interventions [1–5]. While most research has focused on the gut microbiome, variation across and within other body sites has been of growing interest, as evidence of wide-ranging health effects has emerged [6–8]. Large cohort studies of multi-site microbiome data with deep associated metadata and appropriate controls are particularly valuable, especially from whole study populations of all ages and both sexes. Such datasets are hard to come by for humans, and are complicated by multiple factors, including our long lifespans, heterogeneity in consent and other sample access issues, and because of socio-economic confounds. As such, studies of humans have tended to focus on a specific component of the lifespan, such as infanthood, or studies of the elderly, rather than looking at variation across a whole population. An alternative option is to use animal models, such as non-human primates, in which studies of the microbiome can take place on all individuals in a population. We here provide such a study for an important animal model, the rhesus macaque (*Macaca mulatta*). We assess variation in the microbial population of multiple body regions for all individuals of an entire social group of a free-ranging population. In doing so, we focus on the differences in community diversity, taxonomic composition, and function between major life stages—transitions from infancy to juvenescence, from juvenescence to adulthood, and from prime adult years to old age.

During the transition from infanthood, and milk-based to solid food diets, microbial communities are thought to be especially critical. An infant’s microbiome is first seeded during delivery with vaginal microbes from the mother, and is largely uniform across body sites [9], but begins to differentiate within days after birth [10]. After birth, an infant’s gut microbiome is initially dominated by *Bifidobacterium* [11] and *Bacteroides* [12], which contain strains of bacteria known to digest milk oligosaccharides [13, 14]. Among humans, this dominance slowly decreases over the first year of life, while the overall diversity of the gut microbiome increases and reaches adult levels around age 3 [11, 15, 16]. With the addition of solid food and, especially, cessation of breastfeeding, the taxonomic composition of the gut microbiome also transitions to an adult composition [15, 17, 18]. Bacteria that digest plant polysaccharides and fibers, such as in the phylum *Bacteroidetes,* and *Ruminococcus* species, become more abundant [12, 15], along with genes involved in the digestion of complex sugars and starches [19]. The initial seeding of the microbiome during vaginal delivery provides an important base for health in infancy and later in life [18, 20, 21], but how and when disturbances lead to downstream health problems remains an active area of inquiry [22, 23].

While the gut microbiome has received the most attention, the development of the oral microbiome is also of interest, because of its relationship to oral and dental health [24, 25], but there have been few longitudinal studies [7]. Children’s oral microbial diversity increases and changes rapidly after birth [26], and continues to change with the eruption of the deciduous and permanent teeth [24]. Microbial changes related to the eruption of teeth are of special interest because tooth decay may be the most widespread human disease [27]. Studies of children with and without caries have found higher abundance of *Streptococcus mutans* in affected children [28] and higher abundance of *Streptococcus cristatus* in young children has been linked to tooth decay later in childhood [7], while *Porphyromonas catonoiae* and *Neisseria flavescens* were more abundant in caries-free children [25]. However, drawing conclusions on cause and effect from studies of human children is complicated by the confounding effects of diet and oral hygiene; influences that differ widely and for which it is difficult to control. Interestingly, while the oral microbiome is at least partly heritable, this does not seem to include taxa associated with caries propensity [29]. A better understanding of age-related variation in the oral microbiome, especially in pedigreed individuals, may reveal marker species of disease or species that provide a protective function [30].

Another key life transition that is important for understanding human health is advanced aging. Elderly individuals face a host of health challenges, including inflammation, rising susceptibility to infection, constipation, malnutrition, failing dentition, and frailty [31–33]. Given demographic shifts and aging populations in many industrialized countries, such disorders place a significant burden on healthcare systems worldwide and are priorities for research [34, 35]. Changes in gut microbial composition may impact gut epithelium function, cause “inflamm-aging” [36], and contribute to muscle wasting and frailty [3, 37, 38]. The aging gut microbial communities of humans and mice are broadly characterized by a reduction in microbial diversity, a decrease in bacteria in the phyla *Firmicutes* (including in the families *Lachnospiraceae* and *Clostridiaceae*) and *Actinobacteria*, and an increase in *Rikenellaceae* and *Proteobacteria* [37, 39–42]. Such shifts reduce the abundance of short-chain fatty acid producers and increase the abundance of facultative anaerobes, changes which are likely to lead to inflammation [43]. On a functional level, changes in the abundances of creatinine-, carbohydrate-, and lactate-utilizing gut bacteria may negatively impact the digestion of dietary carbohydrates and/or lead to lactate accumulation, which has been linked to inflammatory bowel diseases [39]. Aging-related changes in the oral microbiome have been of interest for reasons beyond oral and dental health [44]. Oral bacteria have been shown or suggested to be directly or indirectly involved in a wide range of diseases that affect the elderly, including cardiovascular issues [45, 46], Alzheimer’s [47, 48], and non-oral cancers [49, 50]. Compared to childhood caries, microbial changes related to tooth decay in the elderly have been understudied, perhaps because of the prevalence of dentures in old age, and more research is needed [51]. With a growing understanding of the microbes involved in age-related health problems, targets for intervention may be identified [41].

A critical factor that needs to be considered in studies of variation across the lifespan is sex as a biological variable [5, 52, 53]. For aging research specifically, this includes the understanding that males and females may have different aging trajectories [54–57], including in key systems like the digestive tract. For example, sex hormones and the gut microbiome may interact to predispose women to autoimmune diseases [58–60] and dietary interventions have been shown to have sex-specific effects on gut microbiota [61]. Effects may also only negatively impact one sex and this may be missed by studies only examining a single sex, which has traditionally tended to be males [62]. This includes the oral microbiome, variations in which may lead to adverse pregnancy outcomes, such as preterm birth, through mechanisms that are not yet fully understood [63]. The need to consider sex differences may be most obvious for research into the genital microbiome, as male and female sex organs present extremely different environments, serve particular functions, and undergo distinct changes with age. However, little is known about how genital microbiomes are established [64] and studies of age-related microbial changes in the genitals have, so far, focused exclusively on women, with a special focus on menopausal changes [8, 65, 66]. How other age-related events affect the vaginal microbiome, such as advent of puberty, first sexual intercourse [67], and advanced age is not well-understood. With the exception of studies related to the effects of circumcision [68] and links to sexually transmitted diseases [69, 70], little is known about how the penile microbiome varies with age and sexual activity [71]. A growing problem in recent decades has been an increase in sexually transmitted infections among the elderly, as the prolonged human lifespan and medical innovations have also led to a prolonged period of sexual activity [72]. However, little is known about how potential microbial changes in the genital microbiomes of elderly men and women may influence transmission risks.

Insight into how the microbiome varies across the lifespan in males and females might ideally come from studies of all individuals within a study group or population, but such studies are difficult in humans for multiple reasons. These include ethical and practical difficulties in enrolling large study cohorts for long-term research, further complicated by the long lifespan of humans. Furthermore, confounding factors, including medical interventions, diet, and socio-economic circumstances, make it difficult to tease apart the various interactions between age-related health and microbial changes. Non-human model organisms provide numerous advantages and have yielded extremely useful data [39, 73–75]. Unfortunately, the major differences in anatomy, physiology, and social biology between humans and most animal models, such as *C*. *elegans*, mice, and rats, impede direct transfer of the information gained to human health advances [76, 77]. Non-human primates offer particularly well-suited model systems for studies in the health sciences [77–79]. Rhesus macaques are perhaps the most important non-human primate model organism for medical research. There are a number of important differences between rhesus macaques and humans, including differences in diet [80], aspects of life history (e.g., an apparent absence of adrenarche in rhesus macaques [81], but see [82]), and the mating system [83]. Nonetheless, rhesus macaques have a relatively close evolutionary relationship with humans, and exhibit generally similar age-related changes in physiology, cognition, and immune function, but on a timescale that is compacted into a 3-4 times shorter lifespan [84, 85]. An additional advantage of using nonhuman primate models is the ability to eliminate the influence of human-specific factors such as access to healthcare, allowing relationships between the microbiome and age and sex to be investigated in the absence of such confounds. However, with some exceptions [86], research on microbial changes in non-human primates across the full lifespan has so far been mostly limited to captivity [87, 88]. Captivity has been demonstrated to measurably change the microbiome of captive primates compared to free-living counterparts [89], underscoring the importance of evaluating findings from captive animals in free-ranging systems. However, studies of free-ranging primates have in turn been largely limited to the measurement of the microbiome from non-invasively collected fecal samples, as such animals are typically not trapped for measurement (e.g., [90–93]). A free-ranging population, like the one studied here, allows for the collection of more invasive sampling, such as annual blood collection, while retaining much of the natural variability and many of the challenges faced by wild populations. The monkeys at this site are provisioned with commercial monkey chow, which may cause differences to reproductive rates and population structure relative to wild populations, but they also feed naturally on other foods found on the island [80]. Importantly, they exhibit natural social behavior, including kin-structured close bonds between females, which are linked to female survival in this population [94], mirroring relationships seen between sociality and survival in wild baboons [95].

Here, we present the first dataset of microbial composition of multiple body sites from free-ranging rhesus macaques from an entire social group living in the same social and ecological environment, including all ages, from 1 month old infants to elderly individuals of 26 years old, and from both males and females. Our aims were to examine (1) microbial community diversity among age and sex classes; (2) taxonomic composition among age and sex classes; and to (3) evaluate potential functional outcomes of microbial diversity and structural variation across age and sex. By examining the relationships between major life stages and the microbiome in free-ranging monkeys, we provide insight into the suitability of macaques as a model for the human microbiome across the lifespan.

## Methods

### Study population

The rhesus macaques sampled for this study are part of the free-ranging population living on the island of Cayo Santiago, Puerto Rico. The island, located off the east coast of Puerto Rico, is 37.5 acres in area and uninhabited except for the macaque colony. From 409 wild-caught Indian rhesus macaques that were released on the island in 1938 [96], the population had grown to ~1700 individuals at the time of this study, which distribute themselves into 7-9 multi-male, multi-female social groups. The monkeys are provisioned with commercial monkey chow and water daily, and supplement their diet with wild foods [80]. This population has been continuously studied since 1956 and genetic samples have been collected from all individuals since 1992 [97]. The macaques mate seasonally, with births clustered in early spring. Most Cayo Santiago females produce their first birth between 3-4 years of age [98], and while males begin mating at 3-4 years, many do not successfully sire offspring until 7-8 years of age [99].

### Sample collection and data generation

We sampled one entire social group, representing animals of all ages and both sexes, which were trapped and anesthetized as part of a colony management plan over the course of 8 weeks from mid-October to mid-December 2016 [100]. We collected one rectal swab and one buccal (oral) swab from each of 105 individuals (aged 1 month-26 years, 64 females, 41 males), and one genital (vaginal or penile) swab from 94 of the same individuals (Table 1; Suppl. Data) by gently inserting a swab into the orifice and rubbing against mucous membranes. For genital samples, the swab was either inserted between the prepuce and glans of the penis (males), or into the vaginal orifice after cleaning the surrounding labial tissue (females) with isopropyl alcohol. We did not sample most of the youngest females because their vaginal orifices were too small to fit the swabs. We used rectal swabs rather than fecal samples as a proxy for the gut microbiome, because rectal swabs could be more reliably collected from all of the individuals in our study. Rectal swabs and fecal samples from the same individual have been shown to be very similar and even interchangeable for inferring gut microbial composition with 16S sequencing [101–103].

**Table 1:** Samples collected for males and females in each age group

Age group	Sex	Body site		
		***Rectal***	***Oral***	***Genital***
Infant (≤1 year)	*Female*	12	11	2
	*Male*	9	9	9
Juvenile (1-4 years)	*Female*	12	12	11
	*Male*	12	12	12
Young adult (5-9 years)	*Female*	19	19	19
	*Male*	10	10	10
Mid-aged adult (10-14 years)	*Female*	14	14	14
	*Male*	5	6	6
Old adult (≥15 years)	*Female*	7	7	7
	*Male*	5	5	4

Samples were stored at −80 °C until they were transported on dry ice to New York University for DNA extraction. We extracted DNA using the DNeasy PowerSoil Kit (Qiagen). We amplified the 16S V4 rRNA region for all samples, along with four extraction blanks, and three dilutions of a mock community using established primers [104] and protocols for dual-indexed libraries [105]. The pooled amplicons were sequenced on an Illumina MiSeq with 2 × 250 bp paired-end sequencing at the University of Minnesota’s Genome Core. Reads were filtered for quality, trimmed to remove barcodes, indices, and primers, and truncated to remove lower quality ends with DADA2 [106].

We assigned amplicon sequence variants (ASVs) and estimated taxonomy with the DADA2 pipeline [106] using the SILVA 16S database v. 132. We identified and removed potential contaminants with the decontam package in R [107]. We were left with a total of 7,031,991 sequences, with a mean of 22,611 sequences per sample. We further removed any samples that had fewer than 2000 sequences and were left with 103 rectal samples, 90 genital samples, and 104 buccal samples. Across all samples, we observed 5470 ASVs of which 1275 remained after filtering the full dataset to remove rare taxa (present no more than twice and in less than 10% of samples). The median number of ASVs per sample was 206.0 (*Q*1 = 109.5, *Q*2 = 359.5). Post-filtering and removal of *Cyanobacteria* to avoid ASVs from dietary items, we retained 856 taxa in the rectal dataset, 678 taxa in the vaginal dataset, 636 taxa in the penile dataset, and 223 taxa in the buccal dataset. When agglomerating at the genus level, we retained 182, 210, 211, and 83 taxa for rectal, vaginal, penile, and buccal samples, respectively.

To facilitate assessment of variation in microbiome community diversity between macaques of different ages, we binned individuals into five age groups: <1 year old (infants), 1-4 years (juveniles), 5-9 years (young adults), 10-14 years (mid-aged adults), and ≥15 years (old adults) (Table 1). These age classes broadly correspond to those used in previous research on this population [108, 109] and are based on life-history transitions, including pre-weaning, onset of sexual activity, cessation of skeletal growth, and prime age. Individuals that are 15 years and older are considered “aged” individuals in the Cayo Santiago population [108, 109].

### Community diversity among age and sex classes

Alpha-diversity across sample types and for age groups within sample types was calculated with Shannon diversity indices within the phyloseq package in R [110]. We assessed community structure (beta-diversity) with Bray-Curtis dissimilarity ordinated with non-metric multidimensional scaling (NMDS). We assessed differences in beta-diversity between sample type, and between different age groups within each sample type with PERMANOVA tests using 9999 permutations in the R package vegan [111]. To identify whether one age group was driving any significant differences in beta-diversity, we followed these tests with pairwise comparisons (PERMANOVA), using the R package pairwiseAdonis [112]. To assess whether plasticity across age groups varied, we further tested for homogeneity of group dispersions implemented in the “betadisper” and “permutest” functions within the R package vegan.

### Taxonomic composition among age and sex classes

Before tests of relative abundance, we agglomerated ASVs at the genus level with phyloseq. We identified the ten most abundant ASVs for each sample type and tested whether there are differences in relative abundance of these ASVs across all age groups (Kruskal-Wallis), between infants and non-infants (all older age classes), and between old macaques (≥15 years old) and all younger age classes (Wilcoxon). For genital samples, we conducted separate vaginal and penile analyses. Because of the low sample size for infant vaginas (*n* = 2), the two youngest age brackets (infants and juveniles) were grouped for the vaginal analyses. We further looked for ASVs that were differentially abundant across age groups, followed by pairwise comparisons of adjacent age groups for each sample type. For this, we identified ASVs with a log2 fold abundance difference greater than +/−2 with a Benjamini–Hochberg adjusted false discovery rate (FDR) *α* value of 0.01 using likelihood ratio tests in the DESeq2 package in R [113].

### Functional outcomes of microbial diversity and structural variation

To assess the potential functional consequences of age differences in community diversity and taxonomic structure, we predicted metagenomic diversity from 16S reads with PiCrust2 [114] and annotated the output with KEGG Brite descriptions at levels 2 and 3. PiCrust2 is an improvement of the original PiCrust software [114] and recent papers have found congruence between PiCrust2 predictions and shotgun sequencing data [114, 115], especially for human data [116]. Functional predictions made by PiCrust2 were parsed and analyzed with DESeq2 and the package FunkyTax [117]. Predicted functions (KEGG genes) were classified as enhanced (frequency of function differs among groups but contributing community does not), divergent (frequency of function and contributing community differs among groups), conserved (frequency of function and contributing community does not differ among groups), or equivalent (frequency of function does not differ among groups but contributing community differs) across age groups with FunkyTax, following methods described previously [117, 118]. We used PERMANOVA to test whether abundances of predicted pathways differed by age group and/or sex using the adonis function in the R package vegan [111] and pairwise adonis tests [112]. For body sites showing evidence of age or sex differences, we further identified KEGG pathways that explain the observed differences between predicted functional profiles with the linear discriminant analysis effect size (LEfSe) method [119], using an LDA effect size cut-off of ≥ 2 and an alpha of 0.01 for both the initial Kruskal-Wallis sum-rank test and the subsequent Wilcoxon rank-sum test.

## Results

### Community diversity among age and sex classes

**Fig. 1:**
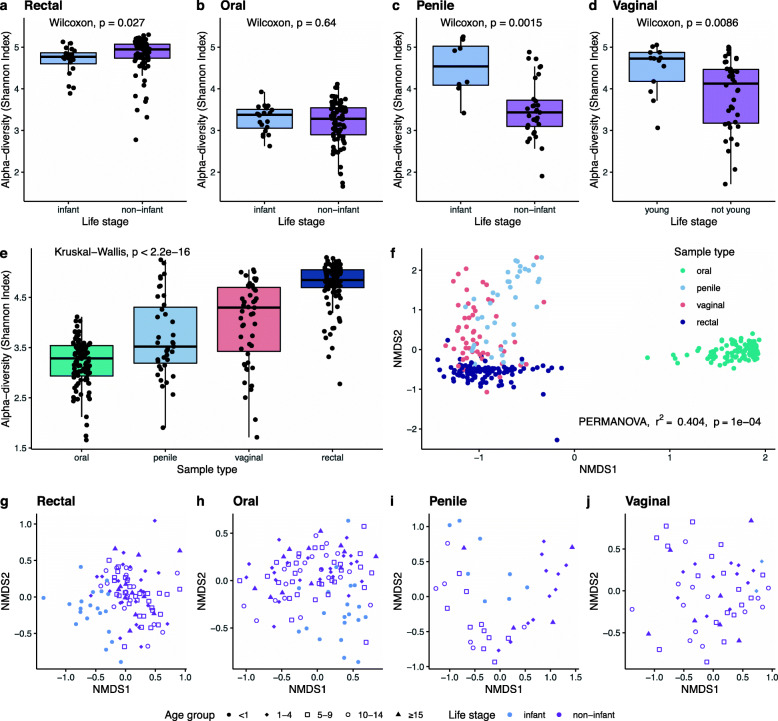
Community diversity in the microbiome of rhesus macaque rectums, oral cavities, penises, and vaginas. Alpha-diversity (Shannon Index) varies between (**a**-**d**) infants and non-infants and (**e**) across sample sites. Beta-diversity varies (**f**) across sample sites and (**g**-**j**) between age groups, with differences driven by infants vs. non-infants in (**f**) rectal, (**g**) oral, and (**i**) penile samples, but (**j**) no age differences in vaginal samples

We find that, when there is significant variation in alpha-diversity, age explains most of the observed variation within each of the three body sites (rectum, oral cavity, and genitals, Fig. 1a-d). Body sites differed significantly from each other in their microbial alpha-diversity (Kruskal-Wallis, *p* < 2.2e−16; Fig. 1e). The alpha-diversity (Shannon Index) of infant rectal samples, the most diverse body region sampled (Fig. 1e), was significantly lower than rectal samples from other age classes (Wilcoxon, *p* = 0.022; Fig. 1a, Fig S1). The oral microbiome had the lowest alpha-diversity of all samples (Fig. 1e) and there were no significant differences between age groups in the alpha-diversity of the oral cavity (Fig. 1b, Fig S1). Infant genitals had higher alpha-diversity than non-infant genitals, both in males (Shannon Index, Wilcoxon, *p* = 0.002; Fig. 1c, Fig S1) and females (Wilcoxon, *p* = 0.009; Fig. 1d, Fig S1). Alpha-diversity did not vary by sex for any of the body sites (Fig S2) and did not vary significantly among other age classes (Fig S1) or with old age (Fig S3).

Community structure (beta diversity) of microbes varied among the sampled body sites (PERMANOVA, *r*^2^=0.404, *p* < 0.001; Fig. 1f) and with age. Age group explained 11.8% (PERMANOVA, *p* = 0.0001), 12.49% (PERMANOVA, *p* = 0.0001), 28.12% (PERMANOVA, *p* = 0.0001) of the variation in beta-diversity for rectal, oral, and penile regions, respectively (Fig. 1g-i). Pairwise comparisons of beta diversity by age group revealed that these differences are driven by the infant (<1 year old) age group (Fig. 1g-h, Table S1, Table S2) in rectal and oral samples. Post-hoc pairwise comparisons of penile beta diversity showed significant differences of 1-4-year-olds to other age groups (Table S3). Beta-diversity of vaginal samples did not vary significantly with age (*r*^2^ = 0.076, *p* = 0.1; Fig. 1j). Results of the homogeneity of dispersion tests also showed that the composition of the penile microbiome was plastic (PERMDISP2, *F* = 2.92, *p* = 0.42) across age groups, while composition of vaginal samples was not (*F* = 1.8, *p* = 0.155). There were no significant sex differences in either rectal or oral samples, but sex clearly distinguished penile and vaginal samples (PERMANOVA, *r*^2^ = 0.107, *p* = 0.0001), which is also reflected in the clear visual separation in the plotted ordination of combined genital samples (Fig. S4).

### Taxonomic composition among age and sex classes

**Fig. 2:**
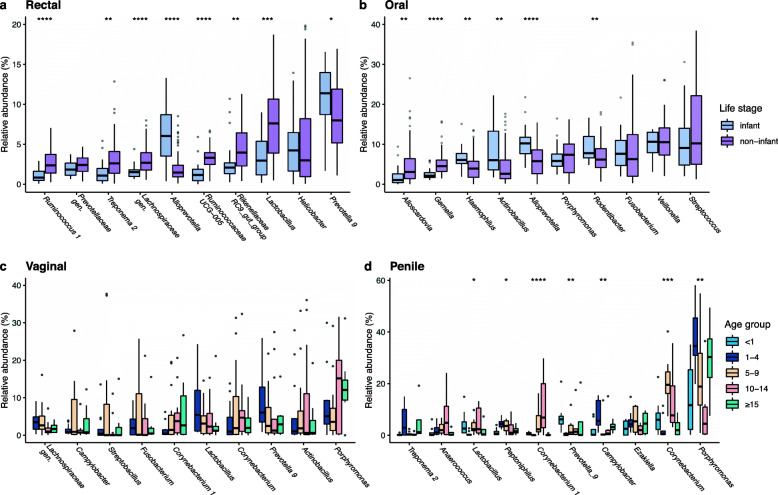
Differences in relative abundances of the top 10 genera were driven by differences between infants and non-infants in (**a**) rectal and (**b**) oral microbiomes. Top 10 genera did not vary by age in (**c**) vaginal samples but did in (**d**) penile microbiomes. The youngest two age groups are pooled for vaginal samples because of small sample sizes for infant vaginas

We find differences in the taxonomic composition of the rectal, oral, penile, and vaginal microbiomes in macaques of different ages. Of the top ten genera in each sample type (Table S4), several differed in their relative abundance across age groups (Fig. S5a, b); however, in the rectum and mouth these differences were driven by the relative abundances observed in infants (Fig. 2a-b), rather than differences between other age groups, including old individuals (Fig. S5c, d). In rectal swabs, six of the top ten most abundant genera were significantly more abundant in non-infants than in infants, including *Lactobacillus* (padj=0.0002), *Rikenellaceae* RC9 gut group (padj=0.005), *Ruminococcaceae* UCG-005 (padj=5.3e−06), *Lachnospiraceae* gen. (padj=9e−05), *Treponema* (padj=0.002), and *Ruminococcus* (padj=4.4e−05). Only *Prevotella_9* (padj=0.032) and *Alloprevotella* (padj=2.1e−05) were higher in infant than non-infant rectums (Fig. 2c). In buccal swabs, four of the ten most abundant taxa were significantly more abundant in infants than non-infants, including *Rodentibacter* (padj=0.005), *Alloprevotella* (padj=0.0003), *Actinobacillus* (padj=0.004), and *Haemophilus* (padj=0.004). *Gemella* (padj=0.0003) and *Alloscardovia* (padj=0.004) were both more abundant in non-infants than in infants (Fig. 2d). Analyses of the top ten ASVs in male and female genital communities revealed age-related differences in the penile community, but not the vaginal community. None of the top ten vaginal genera differed significantly across age groups (Fig. 2c), while seven of the top ten penile genera did, including *Campylobacter* (padj = 0.005), two *Corynebacterium* (both padj < 0.001), and *Prevotella* (padj = 0.004). Unlike the results for rectal and oral communities, age differences in the top ten penile genera are not mainly driven by the infant age group, but also vary across older age groups (Fig. 2d).

The microbiomes of all four body sites are dominated by bacteria in the phyla *Firmicutes* and *Bacteroidetes*, but begin differentiating with the third most abundant taxon. *Proteobacteria* (mean RA = 19.68% ± 2.42%) in the oral cavity, *Actinobacteria* in the penis (20.6% ± 2.92%) and vagina (14.42% ± 3.88%), and *Epsilonbacteraeota* (9.45% ± 4.43%) in the rectal community (Fig. S6). Males and females differ widely in the taxonomic composition of their genital (penile and vaginal, respectively) microbes. Specifically, *Actinobacteria* and *Firmicutes* were significantly more abundant in males, while *Fusobacteria* and *Proteobacteria* were more abundant in females (Fig. S7). Within the rectal community, we do not find age differences in relative abundances of the phyla *Proteobacteria*, *Firmicutes*, or the ratio of *Firmicutes* to *Bacteroidetes* (Fig. S8).

**Fig. 3:**
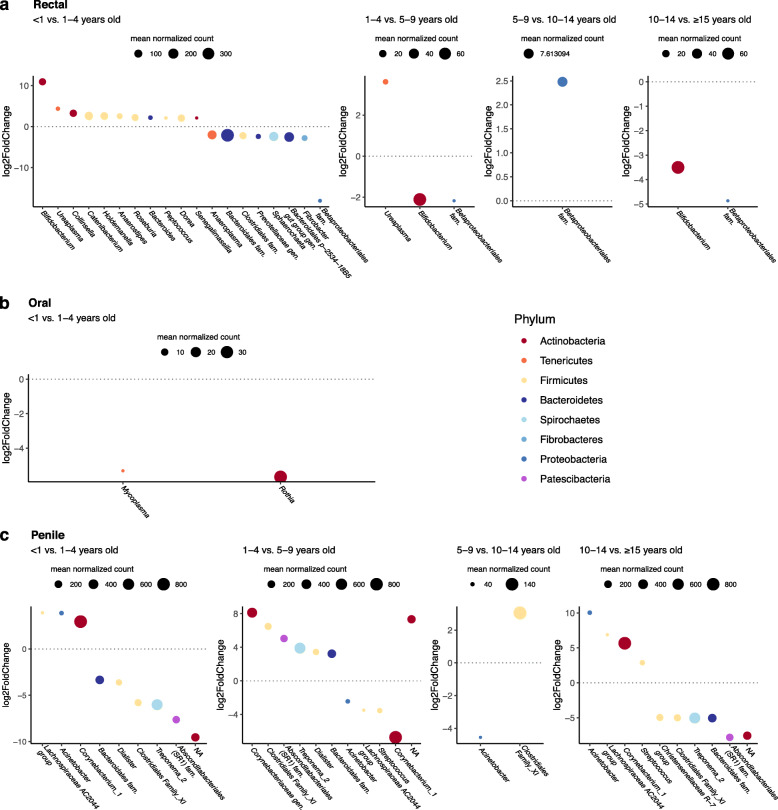
Pairwise comparisons of differentially abundant taxa between age groups in (**a**) rectal, (**b**) oral, and (**c**) penile microbiomes. Figures show bacterial taxa that are significantly differentially abundant between adjacent age groups with a log2fold change of at least +/− 2 (subheadings identify the denominator vs. numerator). NAs reflect bacteria for which order, family, and genus were unidentified. No taxa differed between oral samples after the first age comparison and no taxa differed between vaginal samples in any age group comparison

Across all genera in rectal and oral samples, we find significantly differentially abundant taxa (Suppl. Table S5) especially between age groups <1 and 1-4 years old (Fig. 3a-b). The infant rectum (gut) had significantly more *Bifidobacterium*, *Ureaplasma*, *Collinsella*, *Catenibacterium*, *Holdemanella*, *Anaerostipes*, *Roseburia*, *Bacteroides*, *Dorea*, and *Senegalimassilia*, but less *Anaeroplasma*, *Prevotellacaea gen*., *Sphaerochaeta*, and *Fibrobacter* than guts of 1-4-year-olds (Fig. 3a). Between older age groups, only *Ureaplasma* and *Bifidobacterium* were differentially abundant (Fig. 3a). In the oral community, two fairly low-abundance genera, *Mycoplasma* and *Rothia*, were lower in infants than 1-4-year-olds, but there were no differentially abundant taxa between older age groups (Fig. 3b). In the penile microbiome, *Treponema*, *Lachnospiraceae*, and *Corynebacterium* species are among the more abundant taxa that change in abundance with age (Fig. 3c). No age differences were identified for taxa in vaginal microbiomes.

### Functional outcomes of microbial diversity and structural variation

**Fig. 4:**
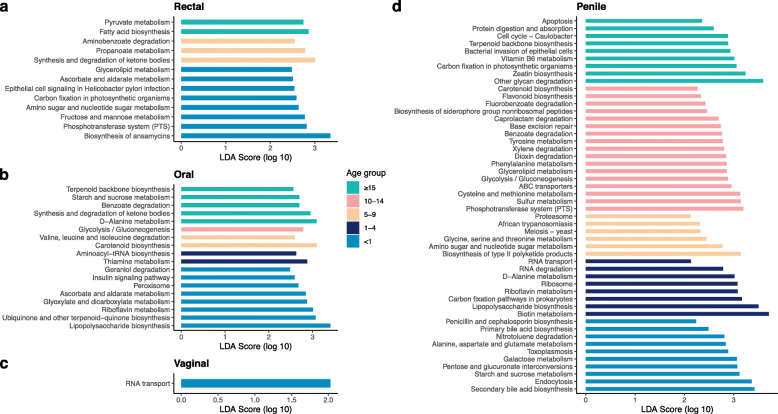
Functional pathways predicted by PiCrust2 that differentiate age groups in (**a**) rectal, (**b**) oral, (**c**) vaginal, and (**d**) penile communities. Differentiating pathways identified by LefSe with LDA effect size ≥2 and alpha ≤ 0.01

To gain further insight into the potential functional effects of the differences in taxonomic structure that we find between age groups, we predicted metagenomic diversity and looked for differential abundance in predicted KEGG genes and pathways. Rectal, oral, and penile samples showed evidence of KEGG genes that were enhanced (frequency of function differs but contributing community does not) and/or divergent (frequency of function and contributing community differs) across age groups, while predicted functions (KEGG genes) of the vaginal microbiome were broadly conserved (frequency of function and contributing community does not differ) (Figure S9). Functional pathways predicted by PiCrust2 differed by age but not sex for rectal (PERMANOVA, *r*^2^ = 0.077, *p* = 0.013) and oral (*r*^2^ = 0.075, *p* = 0.022) communities (Fig. S10a, b). Pairwise comparisons were significant for age groups <1 vs. 5-9 and <1 vs. 10-14 for both body sites (Table S6, Table S7). LEfSe analyses identified 13 (rectal) and 18 (buccal) pathways that differentiate the predicted functional profiles of the five age groups (Fig. 4a, b). Analyses of the genital microbiota revealed strong age effects in the predicted functions of the penile microbiome (*r*^2^ = 0.315, *p* = 0.0001; Fig. S10d), driven by 1-4-year-olds compared to other age groups (Table S8), but no age differences in the predicted functions of the vaginal microbiome (*r*^2^ = 0.075, *p* = 0.2; Fig. S10c). LEfSe identified one pathway in vaginal samples (RNA transport, log 10 LDA score = 2.03, *p* = 0.005, Fig. 4c) and 50 pathways in penile samples (Fig. 4d) that differentiated different age groups. Predicted functional pathways of the genital community varied by sex (*r*^2^ = 0.122, *p* = 0.0001; Fig. S10e) and overall 55 pathways differentiating male and female macaque genital communities were found (Fig. S11).

## Discussion

Our main findings are fourfold: (1) Infant microbial diversity and taxonomic composition are distinct from those of juveniles and other non-infant age groups; (2) diet, e.g., cessation of nursing, appears to be a driver of microbial changes in the rectal and oral communities; (3) the microbiome of male genitals was much more plastic than that of females, especially at the onset of reproductive age; and (4) the differences observed in the gut and oral microbiomes of aged humans relative to other adults are largely absent. Taken together, we observe large changes in the diversity, structure, and predicted function of the microbiome at the transition from infanthood that mimic the patterns seen in humans. However, our results suggest observable differences in the microbiota of aged individuals are largely missing. Below we detail our findings and discuss the implications for rhesus macaques as a model of human microbiome health and function.

### Community diversity differs between infants and non-infants

Comparing species richness and community structure across macaque age groups revealed differences between infants and non-infants, but relative stability across the older age groups, post-infancy, suggesting that the alpha- and beta-diversity of the adult microbiome is largely shaped during the first year of life and robust to perturbations later. In human infants, the alpha-diversity of the gut microbiome is consistently found to be lower than in adults [11, 15]. This is widely interpreted to be due to the introduction of new diversity from food, which increases with the consumption of foods other than mother’s milk [120]. While the population on Cayo Santiago is provisioned with commercial monkey chow, the monkeys also feed on a variety of vegetation and other foods found on the island. These other food sources make up close to half of their dietary intake [80]. Our findings mirror these human studies, and infant rectal samples stand out from all other age classes in having lower alpha diversity (Fig. S1). That this pattern was also found in recent work on captive rhesus macaques [87], suggests a robust and consistent trend. Microbial diversity in the oral cavity was stable across all age groups (Fig. 1a, Fig. S1), suggesting perhaps a limited impact of shifting from milk to solid foods on microbial species diversity in the mouth. In contrast to results of both human and mouse studies [32, 37, 121], we did not observe a decrease in alpha-diversity for any of the body sites in aged macaques.

Interestingly, infant genitals, both penile and vaginal, had higher alpha-diversity, and young male but not female macaques had a different community structure than adults. This pattern of higher alpha-diversity in infants has also been identified for vaginal microbiomes of wild olive baboons [122]. The strong age-effects in the penile community, specifically differences between 1-4-year-olds and other age groups (Table S8), may relate to the onset of sexual behaviors. However, it is surprising that sexual debut does not appear to shift the microbial community structure of female genital microbiomes.

### Taxonomic composition differs between infants and non-infants

As with community diversity, we found that taxonomic composition of rectal and oral microbiomes varied mostly between infants and non-infants (Fig. 2a), but less so in other age groups, and within the genitals, only males showed age-related taxonomic variation. Taxonomic differences between age groups were especially clear in the rectal/gut microbiome. Of the top ten genera, nine differed in their relative abundance in infants. Bacteria previously linked to the digestion of breast milk [12, 123–126], such as *Bifidobacterium*, *Catenibacterium*, and *Bacteroides* were more abundant in infants. *Lactobacillus* also plays an important role in digesting milk and is common in the guts of infants. However, it becomes more abundant post-infancy, possibly because of competition with the infants’ endogenous lactose-digesting enzymes, expression of which decreases with age [127]. Likewise, infants had lower abundances of fiber-degrading bacteria, including in the genera *Fibrobacter*, *Treponema*, and *Ruminococcaceae*, and *Lachnospiraceae*. Variation in relative abundance of *Ruminococcus* (*Ruminococcus champanellensis* and UCG-005), a cellulose-degrading bacteria [128, 129], has previously been linked to transitions to plant foods [130] and were more common in non-infant guts. Our findings on taxonomic differences in infant rhesus macaques are congruent with patterns observed in human infants and children [11, 15, 16], as well as with recent work on captive rhesus macaque infants [87]. Relative abundances of the top ten genera remained largely stable after the first year of life at all three body sites, including into old age.

Unrelated to the age differences discussed here, it is interesting to note that one of the most abundant ASVs in the gut microbiome of free-ranging rhesus macaques is *Prevotella copri*, a bacterium that is prevalent in humans with non-Westernized diets, but which is reduced in populations with diets high in processed foods [131]. Similarly, *Treponema berlinense*, common here, has previously been identified in humans living in rural settings, but was absent from a comparable urban population [132]. Finally, *Helicobacter macacae*, potentially protective against diarrheal disease [87], is highly abundant in macaques of all ages, which may explain why members of this free-ranging population do not commonly exhibit diarrhea [133], while their captive counterparts do [134, 135].

The human and mouse literature on aging gut microbiomes shows consistently that old individuals have a decrease in *Firmicutes* (such as within the families *Lachnospiraceae*, *Clostridiaceae*) and *Bifidobacterium* and an increase in *Rikenellaceae* and *Proteobacteria* [40, 136]. In our population of free-ranging macaques, we did not observe the predicted changes in expression of *Firmicutes* or *Proteobacteria* overall, nor within taxa in *Rikenellaceae*, *Clostridiaceae*, or *Lachnospiraceae*. Of these candidate taxa, only *Bifidobacterium* changed in old macaques; however, it was more abundant in >15-year-olds than 10-14-year-olds (Fig. 3a), not less as expected based on the human and mouse literature [136]. Recent work on a small sample of captive rhesus macaques also did not find human-like age effects on the gut microbiome, although they reported trends for increased *Proteobacteria*, decreased *Firmicutes*, and changes in *Firmicutes*/*Bacteroidetes* ratios with age [88], which we did not confirm with a larger sample of free-ranging macaques (Suppl. Fig. S8).

The oral (buccal) microbiome of free-ranging macaques showed broad similarities to that of humans, including overlap in the prevalent bacterial genera [24–26]. Five of the top 10 most abundant genera that we identified are considered to be part of the healthy human core oral microbiome (*Streptococcus*, *Veillonella*, *Fusobacterium*, *Haemophilus*, and *Porphyromonas* [44, 137, 138]) and a further three are also common in human mouths (*Gemella*, *Alloprevotella*, *Alloscardovia* [44, 139, 140]). However, patterns of taxonomic changes between infants and adults did not always closely match those reported for humans. While human infants have high abundances of *Streptococcus* that decrease with age [7, 26], this genus was equally highly abundant across rhesus macaques irrespective of age. Likewise, *Veillonella* and *Porphyromonas* did not exhibit the age-related changes found in humans [7]. As in humans, however, *Gemella* and *Rothia* increased with age, possibly supported by the additional adhesion surfaces provided by erupting teeth [7]. The taxon *Actinobacillus* (also known as *Aggregatibacter*) was among the most common genera in the macaque oral microbiome, and significantly more abundant in infants. Species in this genus are linked to aggressive periodontitis, which typically affects incisors and first molars of teenagers, as well as to chronic periodontitis [44]. The species *A*. *actinomycetemcomitans*, for example, has been found to be more common in aggressive (juvenile) than adult periodontitis [141] and may serve as a biomarker for disease risk later in life [142, 143]. In elderly humans, disease-associated bacteria, such as *Porphyromonas*, *Treponema*, and *Tannerella* become more abundant in the mouth [32]; however, we did not identify changes in the abundances of these, or any other taxa in aged macaques.

Males and females differed widely in the taxonomic composition of their genital (penile and vaginal, respectively) microbes (Fig. S7). Several of the taxa found to be more abundant in male macaques, such as *Finegoldia*, *Prevotella*, and *Staphylococcus* are also highly abundant in human sperm [144, 145], so may reflect ejaculate remaining in or on the penises of our study subjects. Unlike the human vaginal microbiome, the vaginas of female macaques are not dominated by *Lactobacillus*, supporting previous findings on the comparative vaginal microbiome of primates [146–148]. Captive macaques have been found to have high abundances of species related to bacterial vaginosis, such as *Gardnerella* and *Sneathia* [149–151]. However, these taxa were not found in the vaginal microbiomes of free-ranging rhesus macaque females studied here.

While we did not detect age differences in the vaginal microbiome, the taxonomic composition of the penile microbiome is quite variable across age groups. Unlike other body sites, changes were not limited to differences between infants and non-infant males, however. It is noteworthy that species in the genus *Corynebacterium* were significantly more abundant in adults (5-9 and 10-14-year-olds) than infants, juveniles, or old macaques. This includes *C*. *glucuronolyticum*, a bacteria recently recognized as an opportunistic pathogen and linked to urogenital tract infections in men [152–154]. Unidentified species of *Treponema* that varied with age in male macaque genitals may also warrant further study, as some *Treponema* species are known human and non-human primate pathogens, most notably strains of *Treponema pallidum*, which cause syphilis and yaws in humans and genital ulcers in baboons [155, 156].

### Functional outcomes of microbial diversity and structural variation

In the rectum and mouth, the largest number of variably abundant predicted pathways was identified for the infant age group, rather than other age groups, indicating that variation in community diversity and taxonomic structure translates into functional differences. As with taxonomic differences, diet may be a strong driver of functional changes—many of the predicted functions that differentiate age groups in the gut and mouth are related to metabolism of carbohydrates, lipids, and vitamins (Fig. 4a, b). Interestingly, infant macaques had higher abundances of the pathways “fructose and mannose metabolism,” “ascorbate and aldarate metabolism,” and “amino sugar and nucleotide sugar metabolism,” which may be linked to the digestion of sugars in the milk they consume. While the main disaccharide in milk, lactose, is broken down into monosaccharides by the infants’ endogenous lactase enzymes, recent work in humans has shown that breast milk also contains other sugars [157], including fructose, which is modulated by diet [158]. Because the macaques’ supplemental commercial diet contains sugar, it is likely that their milk also contains non-lactose sugars. The elevated pathway abundances in infants may reflect the microbial digestion both of these sugars and the products of lactase activity. A recent study on longitudinal changes in the salivary microbiome of children found decreases in pathways related to carbohydrate metabolism and increases in xenobiotic degradation over time [159]. Consistent with this, two of the most abundant pathways in infant macaque mouths are glyoxylate and dicarboxylate metabolism and ascorbate and aldarate metabolism, while a top pathway in old macaques is benzoate degradation. In the gut, infants had higher abundances of a pathway related to *Helicobacter pylori* infection, despite *H. pylori* not being present in gut samples, suggesting that this could be a response to another pathogen.

Within the genitals, only the penile microbiome exhibited shifts in predicted functions across age groups, whereas predicted functions of the vaginal microbiome remained extremely stable and conserved across the lifespan. While age was a strong predictor of functional variation in the penile community, at least some of the predicted pathways that vary by age may have been contributed by environmental sources. Macaques in the 10-14-year-old age group were characterized by a large number of pathways that are part of xenobiotics biodegradation, including xylene, benzoate, and dioxin degradation, perhaps reflecting an accumulation of foreign contaminants under the foreskin. The large number of predicted functional pathways identified by LEfSe as differentiating male and female genital communities reflects the very different microbial environments, and accessibility, of the vagina and penis. However, it is surprising that microbial functions of reproductive-age females did not change or become more similar to those of males. This may be due to lack of recent mating, as samples were collected prior to the onset of the mating season. Future work should investigate short-term changes that may arise in the vaginal microbiome as a result of penile microbes introduced during sexual activity.

### Rhesus macaques as a microbiome model for understanding age and sex changes

Our results indicate that rhesus macaques are an excellent model organism for studying gut microbial changes in early life, but their utility for understanding changes in the elderly remains unclear. Developments and transitions in the infant rhesus macaque gut microbiome mirror those of human infants, but similar to findings from captive rhesus macaques [88], we did not find decreased microbial diversity or shifts in abundance of bacterial groups that have been reported for aged humans.

We find that the vaginal microbiota are extremely stable, whereas the penile microbiome is quite dynamic across age groups in both taxonomic composition and predicted microbial functions. This is a seasonally breeding species and rhesus macaques typically start engaging in sexual behavior at age 3-4, so the age-related penile microbiome variation may be related to the onset of and engaging in sexual activity. The lack of age-related taxonomic and functional variation in female genitals is perhaps surprising, as previous research has shown the vaginal microflora to be responsive to changes, including menses [148, 160, 161], sexual debut [67], sexual activity [71, 160], hormonal changes [8, 162–164], and giving birth [165]. However, research on the vaginal microbiome has focused largely on diseases and clinical issues, such as bacterial vaginosis, infertility, and preterm birth, and few studies have directly examined age differences in the human vaginal microbiome. A strength of our study is the highly consistent environment, which minimizes confounding influences of other factors. Our results suggest that selection may have favored the primate vaginal microbiome to be more stable and robust to age and socially related perturbations than the penile microbiome. In this, our results are more consistent with studies finding consistency in vaginal microbiota across the life course. For example, the vaginal microbiota of premenarcheal girls was found to be indistinguishable from those of adult women [166] and a study of olive baboons (*Papio anubis*) similarly found no taxonomic or functional differences in the vaginal microbiome with age or cycle stage [122]. Given that rhesus macaques have a polygynandrous mating system [83], the mechanisms that contribute to vaginal microbial stability, despite exposure to diverse penile microbiota, are remarkable and warrant further examination.

We suggest two possibilities for the apparently discrepant findings for the aged gut and oral microbiome. (1) It is possible that macaques simply do not live long enough to experience the same changes as elderly humans—the extreme longevity seen in modern-day humans is a recent development [167] that is not found in most other species. However, (2) it is also possible that the patterns identified in elderly humans may be due to lifestyle changes or medical interventions, rather than the aging process itself. In this case, rhesus macaques may present a valuable model that is free from these confounding factors. This is especially true for the rhesus macaque population of Cayo Santiago, which live in the same social and ecological environment, and receive a standardized diet, but without medical interventions. An unavoidable limitation of the present study is that we provide a cross-sectional snapshot of an entire social group of rhesus macaques, rather than a longitudinal analysis of the same individuals across the lifespan. We thus cannot exclude the possibility that our findings were influenced by unknown structural differences between the age groups that were unrelated to aging. However, future studies of this population will be able to build on the cross-sectional analyses presented here, by sampling individuals as they age and investigating longitudinal changes of the microbiome within the same individual. Our findings add important data on a previously neglected area of inquiry, age-related effects on the genital microbiome of both sexes, and underscore the utility of rhesus macaques for understanding microbial transitions across multiple body sites.

## Supplementary Information

**Additional file 1: Table S1.** Pairwise adonis results – rectal. **Table S2.** Pairwise adonis results – oral. **Table S3.** Pairwise adonis results – penile. **Table S4.** Top ten genera in rectum, oral cavity, penis, and vagina. **Table S5.** Full results of DESeq2 analysis of differential expression between age groups. **Table S6.** Pairwise adonis results - predicted functional features in rectal community. **Table S7.** Pairwise adonis results - predicted functional features in oral community. **Table S8.** Pairwise adonis results - predicted functional features in penile community. **Figure S1.** Alpha-diversity (Shannon Index) of macaque (a) rectal, (b) oral, (c) penile, and (d) vaginal microbiomes across age groups. **Figure S2.** Alpha-diversity (Shannon Index) of the (a) rectal, (b) oral, and (c) genital microbiomes of males and females. **Figure S3.** Differences in alpha-diversity (Shannon Index) in the (a) rectal, (b) oral, (c) penile, and (d) vaginal microbiomes of macaques younger than 15 years or 15 years and older. **Figure S4.** Beta-diversity of genital samples by sex. Bray-Curtis dissimilarity ordinated with non metric multidimensional scaling (NMDS) shows clear separation of male and female genital samples. **Figure S5.** Differences in relative abundance of top ten genera (a) across all age groups in rectal and (b) oral communities, and between macaques ≥15 years old and <15 years old in (c) rectal and (d) oral communities. The asterisk indicates a significant difference between the groups (a, b: Kruskal-Wallis; c, d: Wilcoxon). **Figure S6.** Average community composition of the rectal, penile, vaginal, and oral microbiomes of free-ranging rhesus macaques. Stacked bars show mean relative abundance of each phylum across all samples for each body site. Phyla in the stacked bars and the legend follow the same order, starting with “<1% abundance” at the right of the bars and ending with “*Tenericutes*” on the left. **Figure S7.** Sex differences in genital microbiome. Figure shows bacterial taxa that are significantly differentially abundant in male genitals vs. female genitals with a log2fold change of at least +/- 2. **Figure S8.** Relative abundances of the phyla (a) *Proteobacteria* and (b) *Firmicutes*, and the (c) ratio of *Firmicutes* to *Bacteroidetes* do not differ across age groups. **Figure S9.** Differences in predicted microbial functions across age groups in (a) rectal, (b) oral, (c) vaginal, and (d) penile communities. Each vertical line is a predicted KEGG gene/function, classified by the CatFun function in FunkyTax as enhanced (frequency of function differs among groups but contributing community does not), divergent (frequency of function and contributing community differs among groups), conserved (frequency of function and contributing community does not differ among groups), or equivalent (frequency of function does not differ among groups but contributing community differs). **Figure S10.** Bray-Curtis distances of predicted functional features of (a) rectal, (b) oral, (c) vaginal, (d) penile, and (e) genital microbial communities ordinated by NMDS. **Figure S11.** Functional pathways predicted by PiCrust2 that differentiate male and female genital communities. Pathways identified by LEfSe with LDA effect size ≥2 and alpha ≤ 0.01.

## Data Availability

Raw sequencing reads are available at the NCBI Sequence Read Archive (SRA) under the accession number PRJNA692377. Metadata used for the analyses are provided as a supplemental data file. All code used during analyses and for generating figures is available via the GitHub repository https://github.com/MareikeJaniak/Cayo16S_Aging_MS.
