# Supplementary material for: Age and sex-associated variation in the multi-site microbiome of an entire social group of free-ranging rhesus macaques
Source: Microbiome. 2021 Mar 22;9:68. doi: 10.1186/s40168-021-01009-w (PMC7986251; doi:10.1186/s40168-021-01009-w)
Supplement: Supplementary file 2 — Additional file 1: Table S1. Pairwise adonis results – rectal. Table S2. Pairwise adonis results – oral. Table S3. Pairwise adonis results – penile. Table S4. Top ten genera in rectum, oral cavity, penis, and vagina. Table S5. Full results of DESeq2 analysis of differential expression between age groups. Table S6. Pairwise adonis results - predicted functional features in rectal community. Table S7. Pairwise adonis results - predicted functional features in oral community. Table S8. Pairwise adonis results - predicted functional features in penile community. Figure S1. Alpha-diversity (Shannon Index) of macaque (a) rectal, (b) oral, (c) penile, and (d) vaginal microbiomes across age groups. Figure S2. Alpha-diversity (Shannon Index) of the (a) rectal, (b) oral, and (c) genital microbiomes of males and females. Figure S3. Differences in alpha-diversity (Shannon Index) in the (a) rectal, (b) oral, (c) penile, and (d) vaginal microbiomes of macaques younger than 15 years or 15 years and older. Figure S4. Beta-diversity of genital samples by sex. Bray-Curtis dissimilarity ordinated with non metric multidimensional scaling (NMDS) shows clear separation of male and female genital samples. Figure S5. Differences in relative abundance of top ten genera (a) across all age groups in rectal and (b) oral communities, and between macaques ≥15 years old and <15 years old in (c) rectal and (d) oral communities. The asterisk indicates a significant difference between the groups (a, b: Kruskal-Wallis; c, d: Wilcoxon). Figure S6. Average community composition of the rectal, penile, vaginal, and oral microbiomes of free-ranging rhesus macaques. Stacked bars show mean relative abundance of each phylum across all samples for each body site. Phyla in the stacked bars and the legend follow the same order, starting with “<1% abundance” at the right of the bars and ending with “Tenericutes” on the left. Figure S7. Sex differences in genital microbiome. Figure shows bacterial taxa that are s [file 40168_2021_1009_MOESM2_ESM.pdf]

Supplemental Tables and Figures

Janiak et al. Age and sex-associated variation in the multi-site microbiome of an entire social group of free-ranging rhesus macaques.

Table S1. Pairwise adonis results - rectal

Pairs (age groups)	Df	SumsOfSqs	F.Model	R2	p.value	P.adjusted (Bonferroni)	sig
5-9 vs 1-4	1	0.2375699	1.1876946	0.02320274	0.228	1.00	
5-9 vs <1	1	1.7293658	8.9764289	0.16036087	0.001	0.01	*
5-9 vs ≥15	1	0.1521490	0.7986834	0.02006809	0.689	1.00	
5-9 vs 10-14	1	0.2290475	1.2192706	0.02582146	0.206	1.00	
1-4 vs <1	1	1.2839041	6.1271395	0.13001297	0.001	0.01	*
1-4 vs ≥15	1	0.1869239	0.8855220	0.02613275	0.616	1.00	
1-4 vs 10-14	1	0.1548619	0.7574719	0.01858486	0.786	1.00	
<1 vs ≥15	1	0.9892766	4.9303748	0.14114864	0.001	0.01	*
<1 vs 10-14	1	1.2281542	6.2839658	0.14517999	0.001	0.01	*
≥15 vs 10-14	1	0.1778731	0.9201401	0.03075320	0.514	1.00	

Table S2. Pairwise adonis results - oral

Pairs (age groups)	Df	SumsOfSqs	F.Model	R2	p.value	P.adjusted (Bonferroni)	sig
5-9 vs 1-4	1	0.07912499	0.5333619	0.01055465	0.844	1.00	
5-9 vs <1	1	1.12275664	8.4124109	0.15181456	0.001	0.01	*
5-9 vs ≥15	1	0.12918209	0.8771434	0.02199614	0.494	1.00	
5-9 vs 10-14	1	0.06938743	0.4816977	0.01014491	0.877	1.00	
1-4 vs <1	1	1.13384122	8.4223219	0.17041534	0.001	0.01	*
1-4 vs ≥15	1	0.13159236	0.8701637	0.02569116	0.478	1.00	
1-4 vs 10-14	1	0.06529297	0.4449099	0.01073497	0.897	1.00	
<1 vs ≥15	1	1.04039755	8.1159586	0.21292810	0.001	0.01	*
<1 vs 10-14	1	1.18951851	9.2774217	0.19623366	0.001	0.01	*
≥15 vs 10-14	1	0.06527936	0.4509110	0.01480780	0.890	1.00	

Table S3. Pairwise adonis results - penile

Pairs (age groups)	Df	SumsOfSqs	F.Model	R2	p.value	P.adjusted (Bonferroni)	sig
5-9 vs 1-4	1	1.7139822	7.997804	0.29623905	0.001	0.01	*
5-9 vs <1	1	0.7717289	3.293306	0.17069684	0.006	0.06	
5-9 vs ≥15	1	0.6375467	2.572801	0.17654814	0.010	0.10	
5-9 vs 10-14	1	0.3041139	1.238296	0.08126213	0.220	1.00	
1-4 vs <1	1	1.2115768	5.089853	0.23041589	0.001	0.01	*
1-4 vs ≥15	1	0.3613845	1.436279	0.09949095	0.178	1.00	
1-4 vs 10-14	1	1.2140584	4.874970	0.24528190	0.002	0.02	.
<1 vs ≥15	1	0.4638427	1.573177	0.13593302	0.115	1.00	
<1 vs 10-14	1	0.5087533	1.788717	0.12972327	0.073	0.73	
≥15 vs 10-14	1	0.5336500	1.618720	0.16828850	0.109	1.00	

Table S4. Top ten genera in rectum, oral cavity, penis, and vagina

Site	ASV	Phylum	Class	Order	Family	Genus
**Rectal**	10	*Firmicutes*	*Clostridia*	*Clostridiales*	*Ruminococcaceae*	*Ruminococcus_1*
	9	*Bacteroidetes*	*Bacteroidia*	*Bacteroidales*	*Prevotellaceae*	*NA*
	8	*Spirochaetes*	*Spirochaetia*	*Spirochaetales*	*Spirochaetaceae*	*Treponema_2*
	7	*Bacteroidetes*	*Bacteroidia*	*Bacteroidales*	*Prevotellaceae*	*Alloprevotella*
	6	*Firmicutes*	*Clostridia*	*Clostridiales*	*Lachnospiraceae*	*NA*
	5	*Firmicutes*	*Clostridia*	*Clostridiales*	*Ruminococcaceae*	*Ruminococcaceae_UCG-005*
	4	*Bacteroidetes*	*Bacteroidia*	*Bacteroidales*	*Rikenellaceae*	*Rikenellaceae_RC9_gut_group*
	3	*Firmicutes*	*Bacilli*	*Lactobacillales*	*Lactobacillaceae*	*Lactobacillus*
	2	*Epsilon-bacteraeota*	*Campylobacteria*	*Campylo-bacterales*	*Helicobacteraceae*	*Helicobacter*
	1	*Bacteroidetes*	*Bacteroidia*	*Bacteroidales*	*Prevotellaceae*	*Prevotella_9*
**Buccal**	10	*Actinobacteria*	*Actinobacteria*	*Bifidobacteriales*	*Bifidobacteriaceae*	*Alloscardovia*
	9	*Firmicutes*	*Bacilli*	*Bacillales*	*Family_XI*	*Gemella*
	8	*Proteobacteria*	*Gammaproteo-bacteria*	*Pasteurellales*	*Pasteurellaceae*	*Haemophilus*
	7	*Proteobacteria*	*Gammaproteo-bacteria*	*Pasteurellales*	*Pasteurellaceae*	*Actinobacillus*
	6	*Bacteroidetes*	*Bacteroidia*	*Bacteroidales*	*Prevotellaceae*	*Alloprevotella*
	5	*Bacteroidetes*	*Bacteroidia*	*Bacteroidales*	*Porphyromonadaceae*	*Porphyromonas*
	4	*Proteobacteria*	*Gammaproteo-bacteria*	*Pasteurellales*	*Pasteurellaceae*	*Rodentibacter*
	3	*Fusobacteria*	*Fusobacteriia*	*Fusobacteriales*	*Fusobacteriaceae*	*Fusobacterium*
	2	*Firmicutes*	*Negativicutes*	*Selenomonadales*	*Veillonellaceae*	*Veillonella*
	1	*Firmicutes*	*Bacilli*	*Lactobacillales*	*Streptococcaceae*	*Streptococcus*
**Penile**	10	*Spirochaetes*	*Spirochaetia*	*Spirochaetales*	*Spirochaetaceae*	*Treponema_2*
	9	*Firmicutes*	*Clostridia*	*Clostridiales*	*Family_XI*	*Anaerococcus*
	8	*Firmicutes*	*Bacilli*	*Lactobacillales*	*Lactobacillaceae*	*Lactobacillus*
	7	*Firmicutes*	*Clostridia*	*Clostridiales*	*Family_XI*	*Peptoniphilus*
	6	*Actinobacteria*	*Actinobacteria*	*Corynebacteriales*	*Corynebacteriaceae*	*Corynebacterium_1*
	5	*Bacteroidetes*	*Bacteroidia*	*Bacteroidales*	*Prevotellaceae*	*Prevotella_9*
	4	*Epsilon-bacteraeota*	*Campylobacteria*	*Campylo-bacterales*	*Campylobacteraceae*	*Campylobacter*
	3	*Firmicutes*	*Clostridia*	*Clostridiales*	*Family_XI*	*Ezakiella*
	2	*Actinobacteria*	*Actinobacteria*	*Corynebacteriales*	*Corynebacteriaceae*	*Corynebacterium*
	1	*Bacteroidetes*	*Bacteroidia*	*Bacteroidales*	*Porphyromonadaceae*	*Porphyromonas*
**Vaginal**	10	*Firmicutes*	*Clostridia*	*Clostridiales*	*Lachnospiraceae*	*NA*
	9	*Epsilon-bacteraeota*	*Campylobacteria*	*Campylobacterales*	*Campylobacteraceae*	*Campylobacter*
	8	*Fusobacteria*	*Fusobacteriia*	*Fusobacteriales*	*Leptotrichiaceae*	*Streptobacillus*
	7	*Fusobacteria*	*Fusobacteriia*	*Fusobacteriales*	*Fusobacteriaceae*	*Fusobacterium*
	6	*Actinobacteria*	*Actinobacteria*	*Corynebacteriales*	*Corynebacteriaceae*	*Corynebacterium_1*
	5	*Firmicutes*	*Bacilli*	*Lactobacillales*	*Lactobacillaceae*	*Lactobacillus*
	4	*Actinobacteria*	*Actinobacteria*	*Corynebacteriales*	*Corynebacteriaceae*	*Corynebacterium*
	3	*Bacteroidetes*	*Bacteroidia*	*Bacteroidales*	*Prevotellaceae*	*Prevotella_9*
	2	*Proteobacteria*	*Gamma-proteobacteria*	*Pasteurellales*	*Pasteurellaceae*	*Actinobacillus*
	1	*Bacteroidetes*	*Bacteroidia*	*Bacteroidales*	*Porphyromonadaceae*	*Porphyromonas*

Table S5. Full results of DESeq2 analysis of differential expression between age groups

Site	Group	Phylum	Class	Order	Family	Genus	base Mean	log2fold Change	lfcSE	stat	padj
rectal	<1 vs. 1-4	*Bacteroidetes*	*Bacteroidia*	*Bacteroidales*	NA	NA	374.110	-2.109	0.394	36.813	3.3E-06
		Spirochaetes	*Spirochaetia*	*Spirochaetales*	*Spirochaetaceae*	*Sphaerochaeta*	150.789	-2.399	0.406	35.690	5.1E-06
		Actinobacteria	*Coriobacteriia*	*Coriobacteriales*	*Coriobacteriaceae*	*Collinsella*	74.808	3.258	0.514	93.260	2.2E-17
		Firmicutes	*Erysipelotrichia*	*Erysipelotrichales*	*Erysipelotrichaceae*	*Holdemanella*	81.686	2.627	0.369	126.743	3.2E-24
		Actinobacteria	*Actinobacteria*	*Bifidobacteriales*	*Bifidobacteriaceae*	*Bifidobacterium*	70.148	10.939	1.389	85.864	5.5E-16
		Firmicutes	*Clostridia*	*Clostridiales*	*Lachnospiraceae*	*Dorea*	66.288	2.121	0.337	85.256	5.6E-16
		Tenericutes	*Mollicutes*	*Anaeroplasmatales*	*Anaeroplasmataceae*	*Anaeroplasma*	139.284	-2.008	0.391	30.593	4.4E-05
		Firmicutes	*Erysipelotrichia*	*Erysipelotrichales*	*Erysipelotrichaceae*	*Catenibacterium*	106.368	2.656	0.903	25.671	3.2E-04
		Bacteroidetes	*Bacteroidia*	*Bacteroidales*	p-2534-18B5_gut_group	NA	177.213	-2.541	0.665	17.884	6.8E-03
		Firmicutes	*Clostridia*	*Clostridiales*	*Lachnospiraceae*	*Roseburia*	56.320	2.264	0.583	42.310	3.0E-07
		Fibrobacteres	*Fibrobacteria*	*Fibrobacterales*	*Fibrobacteraceae*	*Fibrobacter*	34.300	-2.791	0.574	21.339	2.0E-03
		Firmicutes	*Clostridia*	*Clostridiales*	*Lachnospiraceae*	*Anaerostipes*	32.126	2.585	0.706	31.542	3.3E-05
		Firmicutes	*Clostridia*	*Clostridiales*	NA	NA	63.233	-2.169	0.523	18.455	5.4E-03
		Bacteroidetes	*Bacteroidia*	*Bacteroidales*	*Prevotellaceae*	*Prevotellaceae gen.*	21.538	-2.398	0.635	18.968	4.4E-03
		Tenericutes	*Mollicutes*	*Mycoplasmatales*	*Mycoplasmataceae*	*Ureaplasma*	11.330	4.375	1.445	51.510	4.9E-09
		Firmicutes	*Clostridia*	*Clostridiales*	*Peptococcaceae*	*Peptococcus*	6.273	2.132	0.647	17.208	8.7E-03
		Actinobacteria	*Coriobacteriia*	*Coriobacteriales*	*Eggerthellaceae*	*Senegalimassilia*	5.820	2.116	0.773	19.630	3.6E-03
		Bacteroidetes	*Bacteroidia*	*Bacteroidales*	*Bacteroidaceae*	*Bacteroides*	13.274	2.194	0.766	17.017	9.2E-03
		Proteobacteria	*Gammaproteo-bacteria*	*Betaproteo-bacteriales*	NA	NA	7.613	-18.088	1.552	21.135	2.0E-03
	1-4 vs. 5-9	*Actinobacteria*	*Actinobacteria*	*Bifidobacteriales*	*Bifidobacteriaceae*	*Bifidobacterium*	70.148	-2.111	1.316	85.864	5.5E-16
		Tenericutes	*Mollicutes*	*Mycoplasmatales*	*Mycoplasmataceae*	*Ureaplasma*	11.330	3.631	1.374	51.510	4.9E-09
		Proteobacteria	*Gammaproteo-bacteria*	*Betaproteo-bacteriales*	NA	NA	7.613	-2.175	1.362	21.135	2.0E-03
	5-9 vs. 10-14	*Proteobacteria*	*Gammaproteo-bacteria*	*Betaproteo-bacteriales*	NA	NA	7.613	2.481	1.446	21.135	2.0E-03

	10-14 vs. ≥15	*Actinobacteria*	*Actinobacteria*	*Bifidobacteriales*	*Bifidobacteriaceae*	*Bifidobacterium*	70.148	-3.502	1.678	85.864	5.5E-16
		Proteobacteria	*Gammaproteo-bacteria*	*Betaproteo-bacteriales*	NA	NA	7.613	-4.867	1.791	21.135	2.0E-03
buccal	<1 vs. 1-4	*Tenericutes*	*Mollicutes*	*Mycoplasmatales*	*Mycoplasmataceae*	*Mycoplasma*	4.874	-5.311	1.106	24.231	9.3E-04
		Actinobacteria	*Actinobacteria*	*Micrococcales*	*Micrococcaceae*	*Rothia*	32.403	-5.666	0.711	50.922	6.0E-09
penile	<1 vs. 1-4	*Actinobacteria*	*Actinobacteria*	*Corynebacteriales*	*Corynebacteriaceae*	*Corynebacterium_1*	824.833	2.950	1.054	52.982	7.7E-09
		Firmicutes	*Clostridia*	*Clostridiales*	*Lachnospiraceae*	Lachnospiraceae AC2044 group	21.047	3.916	1.211	19.769	4.5E-03
		Patescibacteria	*Gracilibacteria*	*Abscondita-bacteriales_(SR1)*	NA	NA	186.420	-7.630	1.693	25.172	6.9E-04
		Spirochaetes	*Spirochaetia*	*Spirochaetales*	*Spirochaetaceae*	*Treponema_2*	557.906	-6.011	0.937	48.544	3.2E-08
		Actinobacteria	*Actinobacteria*	*NA*	NA	NA	258.686	-9.539	1.818	28.919	1.8E-04
		Bacteroidetes	*Bacteroidia*	*Bacteroidales*	NA	NA	285.037	-3.341	0.884	40.054	1.3E-06
		Proteobacteria	*Gammaproteo-bacteria*	*Pseudomonadales*	*Moraxellaceae*	*Acinetobacter*	38.698	3.871	1.552	26.183	5.2E-04
		Firmicutes	*Clostridia*	*Clostridiales*	*Family_XI*	NA	142.282	-5.777	1.804	22.675	1.5E-03
		Firmicutes	*Negativicutes*	*Selenomonadales*	*Veillonellaceae*	*Dialister*	96.458	-3.591	0.940	20.921	2.9E-03
	1-4 vs. 5-9	*Firmicutes*	*Bacilli*	*Lactobacillales*	*Streptococcaceae*	*Streptococcus*	56.763	-3.525	0.888	19.065	5.7E-03
		Actinobacteria	*Actinobacteria*	*Corynebacteriales*	*Corynebacteriaceae*	*Corynebacterium_1*	824.833	-6.725	0.992	52.982	7.7E-09
		Firmicutes	*Clostridia*	*Clostridiales*	*Lachnospiraceae*	Lachnospiraceae AC2044 group	21.047	-3.481	1.144	19.769	4.5E-03
		Actinobacteria	*Actinobacteria*	*Corynebacteriales*	*Corynebacteriaceae*	NA	424.314	8.119	1.597	22.709	1.5E-03
		Patescibacteria	*Gracilibacteria*	*Abscondita-bacteriales_(SR1)*	NA	NA	186.420	5.042	1.568	25.172	6.9E-04
		Spirochaetes	*Spirochaetia*	*Spirochaetales*	*Spirochaetaceae*	*Treponema_2*	557.906	3.900	0.877	48.544	3.2E-08
		Actinobacteria	*Actinobacteria*	*NA*	NA	NA	258.686	7.335	1.678	28.919	1.8E-04
		Bacteroidetes	*Bacteroidia*	*Bacteroidales*	NA	NA	285.037	3.231	0.832	40.054	1.3E-06
		Proteobacteria	*Gammaproteo-bacteria*	*Pseudomonadales*	*Moraxellaceae*	*Acinetobacter*	38.698	-2.440	1.473	26.183	5.2E-04
		Firmicutes	*Clostridia*	*Clostridiales*	*Family_XI*	NA	142.282	6.504	1.705	22.675	1.5E-03
		Firmicutes	*Negativicutes*	*Selenomonadales*	*Veillonellaceae*	*Dialister*	96.458	3.461	0.886	20.921	2.9E-03

5-9 vs. 10-14	*Proteobacteria*	*Gammaproteo-bacteria*	*Pseudomonadales*	*Moraxellaceae*	*Acinetobacter*	38.698	-4.552	1.676	26.183	5.2E-04
	Firmicutes	*Clostridia*	*Clostridiales*	*Family_XI*	NA	142.282	3.067	2.123	22.675	1.5E-03
10-14 vs. ≥15	*Firmicutes*	*Bacilli*	*Lactobacillales*	*Streptococcaceae*	*Streptococcus*	56.763	2.897	1.314	19.065	5.7E-03
	Actinobacteria	*Actinobacteria*	*Corynebacteriales*	*Corynebacteriaceae*	*Corynebacterium_1*	824.833	5.666	1.451	52.982	7.7E-09
	Firmicutes	*Clostridia*	*Clostridiales*	*Lachnospiraceae*	Lachnospiraceae AC2044 group	21.047	6.904	1.927	19.769	4.5E-03
	Patescibacteria	*Gracilibacteria*	*Abscondita-bacteriales_(SR1)*	NA	NA	186.420	-7.812	2.326	25.172	6.9E-04
	Spirochaetes	*Spirochaetia*	*Spirochaetales*	*Spirochaetaceae*	*Treponema_2*	557.906	-5.037	1.297	48.544	3.2E-08
	Actinobacteria	*Actinobacteria*	NA	NA	NA	258.686	-7.572	2.508	28.919	1.8E-04
	Bacteroidetes	*Bacteroidia*	*Bacteroidales*	NA	NA	285.037	-5.059	1.228	40.054	1.3E-06
	Proteobacteria	*Gammaproteo-bacteria*	*Pseudomonadales*	*Moraxellaceae*	*Acinetobacter*	38.698	10.044	2.320	26.183	5.2E-04
	Firmicutes	*Clostridia*	*Clostridiales*	*Christensenellaceae*	Christensenellaceae R-7 group	140.411	-4.939	1.204	24.800	7.0E-04
	Firmicutes	*Clostridia*	*Clostridiales*	*Family_XI*	NA	142.282	-4.983	2.595	22.675	1.5E-03

Table S6. Pairwise adonis results - predicted functional features in rectal community

Pairs (age groups)	Df	SumsOfSqs	F.Model	R2	p.value	P.adjusted (Bonferroni)	sig
5-9 vs 1-4	1	0.0010306721	0.7769953	0.01530211	0.532	1.00	
5-9 vs <1	1	0.0072127812	6.1328801	0.11542533	0.001	0.01	*
5-9 vs ≥15	1	0.0018513156	1.2045526	0.02996060	0.275	1.00	
5-9 vs 10-14	1	0.0010910491	0.9177349	0.01956051	0.426	1.00	
1-4 vs <1	1	0.0042799811	3.3419498	0.07536768	0.006	0.06	
1-4 vs ≥15	1	0.0016181180	0.9339795	0.02752343	0.399	1.00	
1-4 vs 10-14	1	0.0006638146	0.5114213	0.01262413	0.765	1.00	
<1 vs ≥15	1	0.0041576410	2.7042054	0.08268678	0.026	0.26	
<1 vs 10-14	1	0.0036855949	3.3365290	0.08271731	0.004	0.04	.
≥15 vs 10-14	1	0.0016182615	1.0306191	0.03431894	0.391	1.00	

Table S7. Pairwise adonis results - predicted functional features in oral community

Pairs (age groups)	Df	SumsOfSqs	F.Model	R2	p.value	P.adjusted (Bonferroni)	sig
5-9 vs 1-4	1	0.0016295221	0.8662330	0.017029627	0.458	1.00	
5-9 vs <1	1	0.0069453884	4.7962496	0.092598396	0.002	0.02	.
5-9 vs ≥15	1	0.0009410774	0.4815296	0.012196326	0.744	1.00	
5-9 vs 10-14	1	0.0012980851	0.7022642	0.014721822	0.574	1.00	
1-4 vs <1	1	0.0065479066	4.2132201	0.093185580	0.008	0.08	
1-4 vs ≥15	1	0.0005921717	0.2718689	0.008171133	0.893	1.00	
1-4 vs 10-14	1	0.0020416191	1.0141864	0.024139143	0.355	1.00	
<1 vs ≥15	1	0.0060636311	3.9647844	0.116732213	0.008	0.08	
<1 vs 10-14	1	0.0092378898	6.2089569	0.140445678	0.001	0.01	*
≥15 vs 10-14	1	0.0005139447	0.2383155	0.007881241	0.939	1.00	

Table S8. Pairwise adonis results - predicted functional features in penile community

Pairs (age groups)	Df	SumsOfSqs	F.Model	R2	p.value	P.adjusted (Bonferroni)	sig
5-9 vs 1-4	1	0.017268293	6.113696	0.24344071	0.005	0.05	.
5-9 vs <1	1	0.008561219	2.406054	0.13072079	0.066	0.66	
5-9 vs ≥15	1	0.009060431	2.384076	0.16574411	0.068	0.68	
5-9 vs 10-14	1	0.005526593	1.211777	0.07966046	0.270	1.00	
1-4 vs <1	1	0.028565811	11.074107	0.39445982	0.001	0.01	*
1-4 vs ≥15	1	0.003106345	1.241572	0.08717942	0.287	1.00	
1-4 vs 10-14	1	0.026797842	7.917297	0.34547255	0.002	0.02	.
<1 vs ≥15	1	0.011659878	3.257815	0.24572791	0.056	0.56	
<1 vs 10-14	1	0.005475232	1.215907	0.09200333	0.323	1.00	
≥15 vs 10-14	1	0.015767371	2.953413	0.26963402	0.048	0.48	

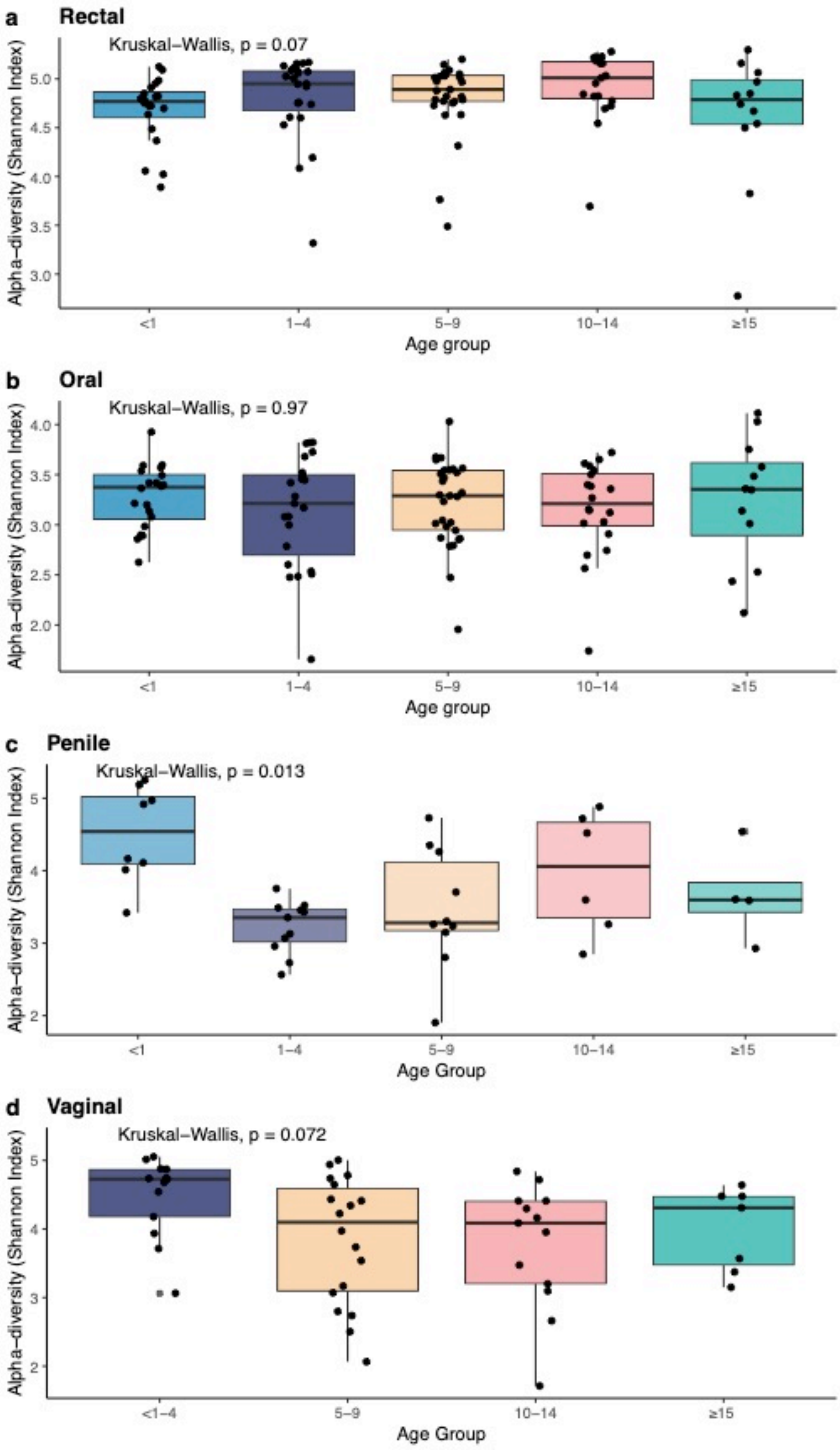

Figure S1. Alpha-diversity (Shannon Index) of macaque (a) rectal, (b) oral, (c) penile, and (d) vaginal microbiomes across age groups.

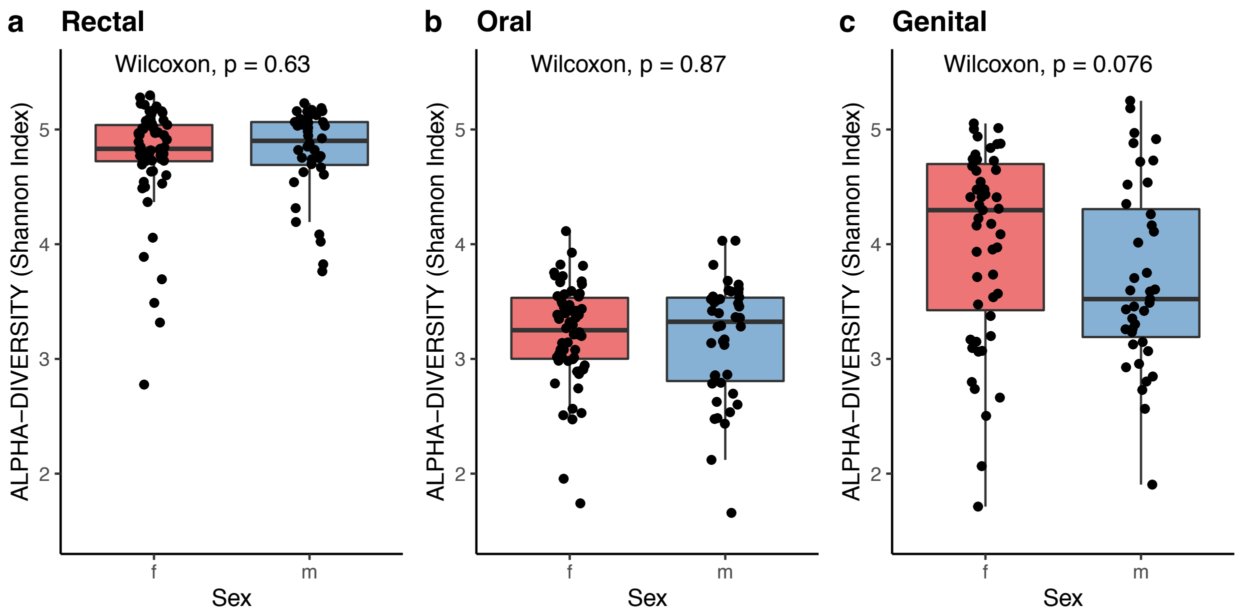

Figure S2. Alpha-diversity (Shannon Index) of the (a) rectal, (b) oral, and (c) genital microbiomes of males and females.

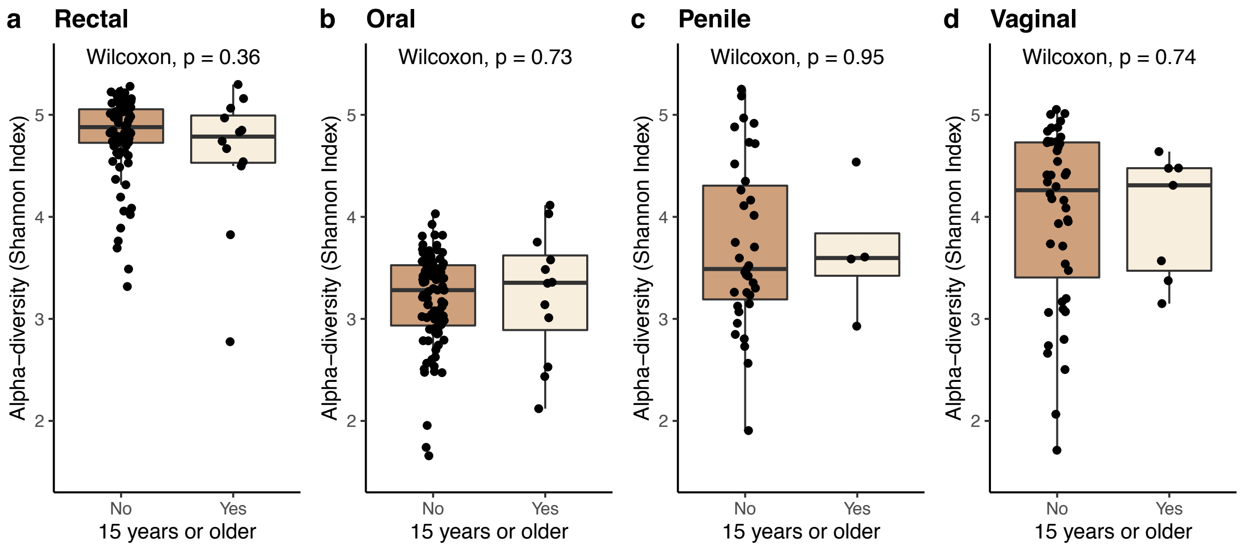

Figure S3. Differences in alpha-diversity (Shannon Index) in the (a) rectal, (b) oral, (c) penile, and (d) vaginal microbiomes of macaques younger than 15 years or 15 years and older.

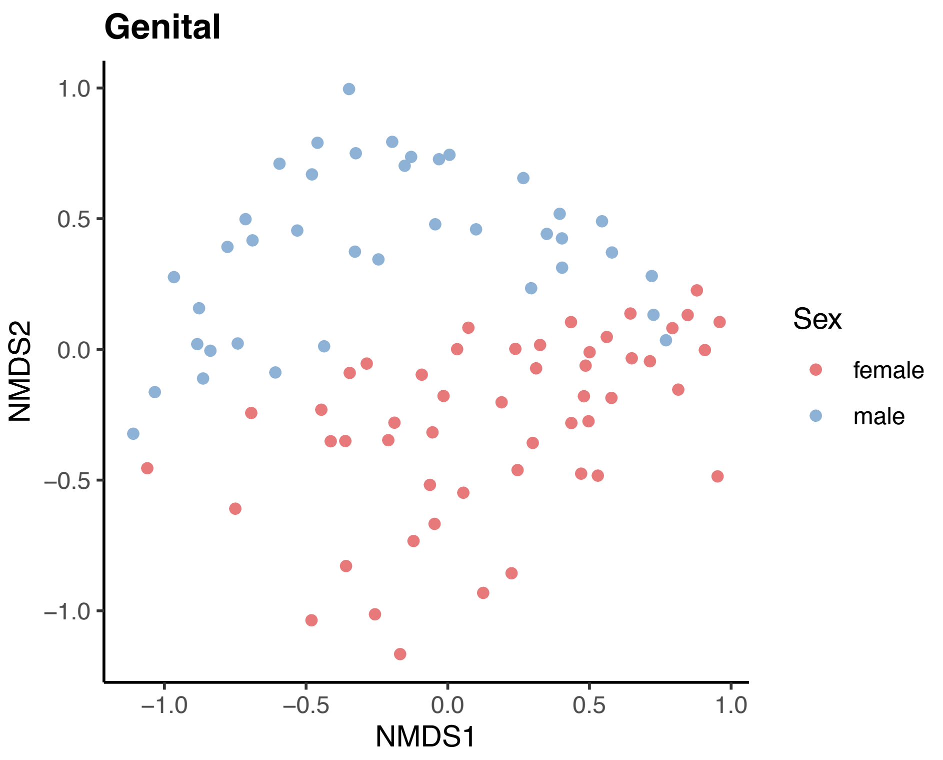

Figure S4. Beta-diversity of genital samples by sex. Bray-Curtis dissimilarity ordinated with non-metric multidimensional scaling (NMDS) shows clear separation of male and female genital samples.

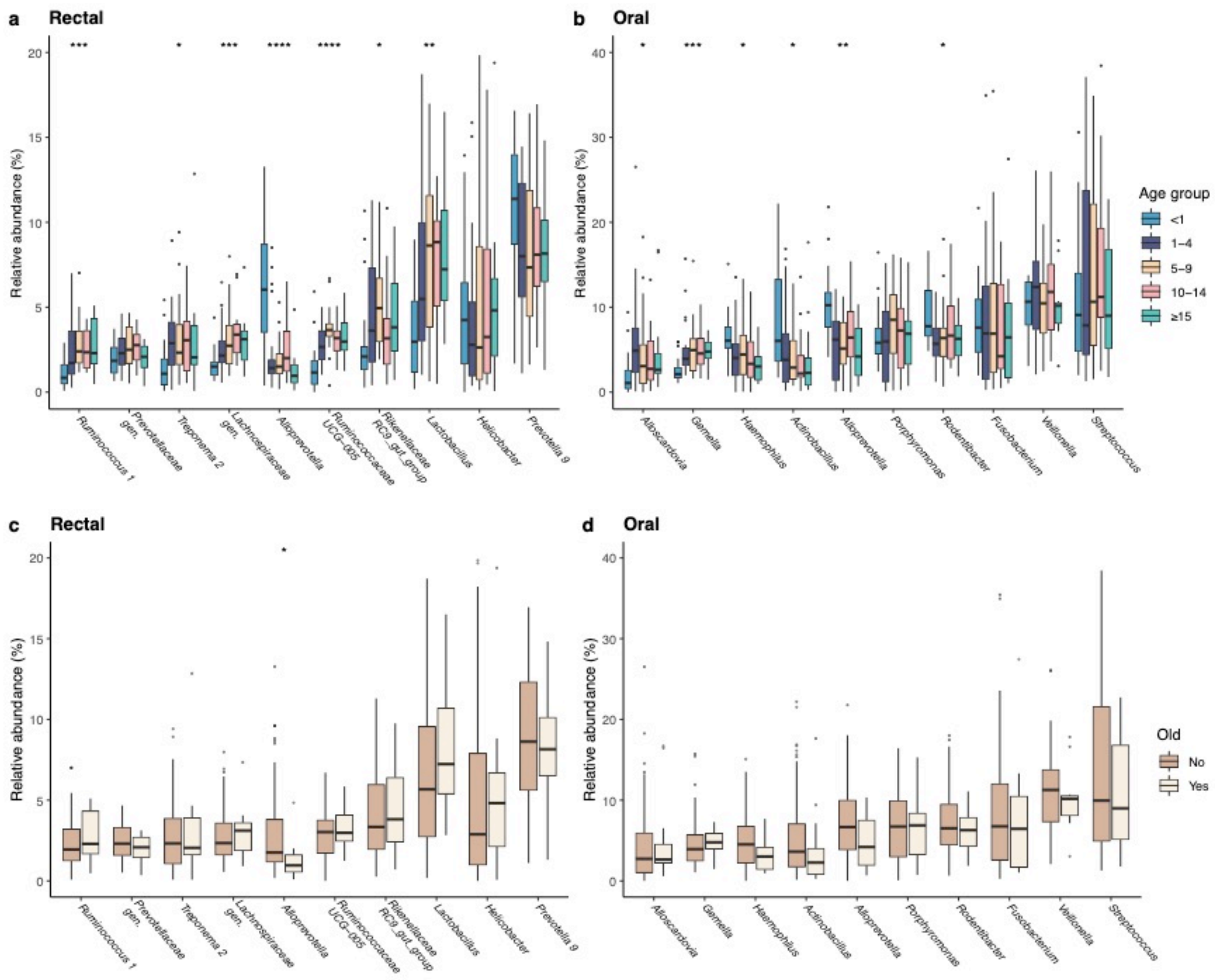

Figure S5. Differences in relative abundance of top ten genera (a) across all age groups in rectal and (b) oral communities, and between macaques ≥15 years old and <15 years old in (c) rectal and (d) oral communities. The asterisk indicates a significant difference between the groups (a, b: Kruskal-Wallis; c, d: Wilcoxon).

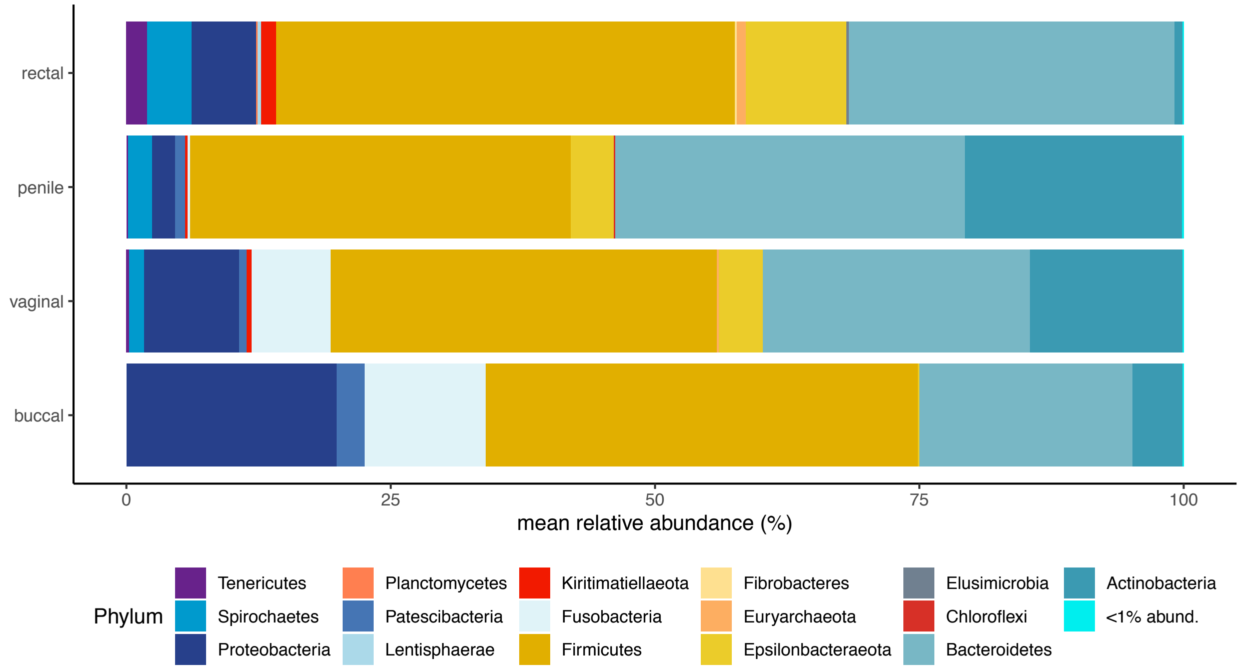

Figure S6. Average community composition of the rectal, penile, vaginal, and oral microbiomes of free-ranging rhesus macaques. Stacked bars show mean relative abundance of each phylum across all samples for each body site. Phyla in the stacked bars and the legend follow the same order, starting with "<1% abundance" at the right of the bars and ending with "*Tenericutes*" on the left.

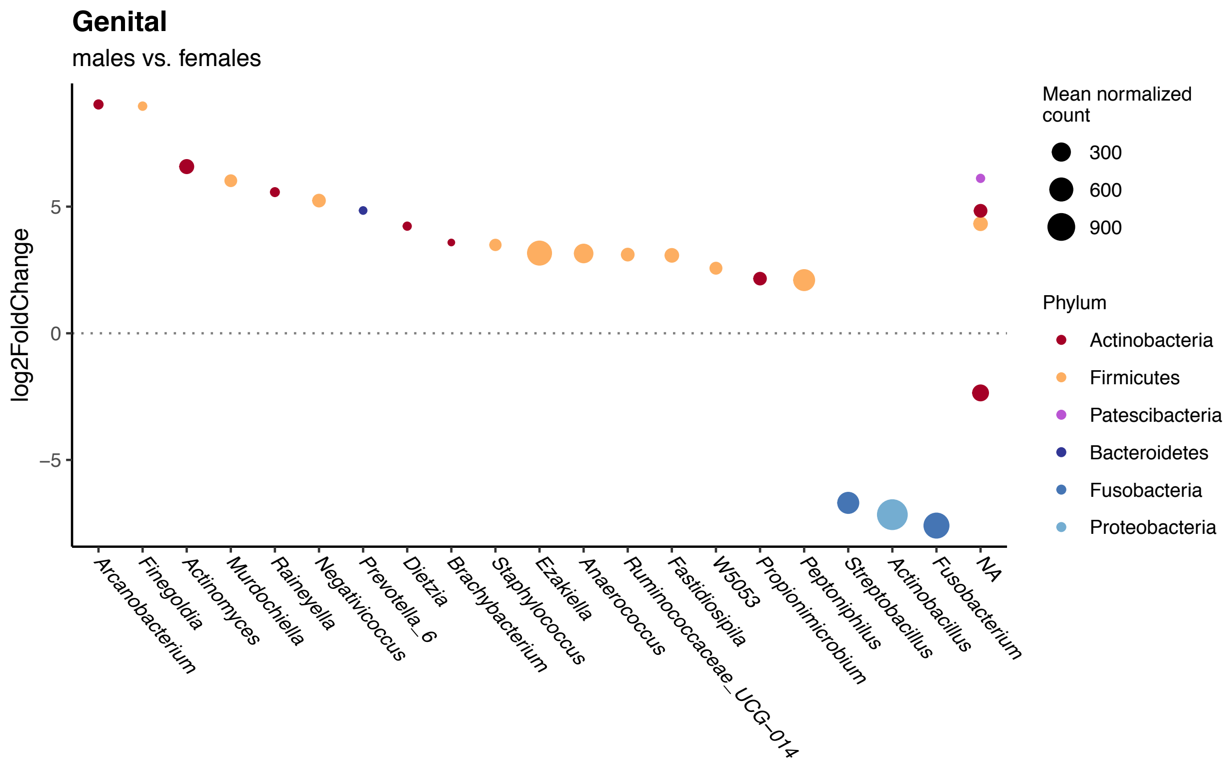

Figure S7. Sex differences in genital microbiome. Figure shows bacterial taxa that are significantly differentially abundant in male genitals vs. female genitals with a log2fold change of at least +/- 2.

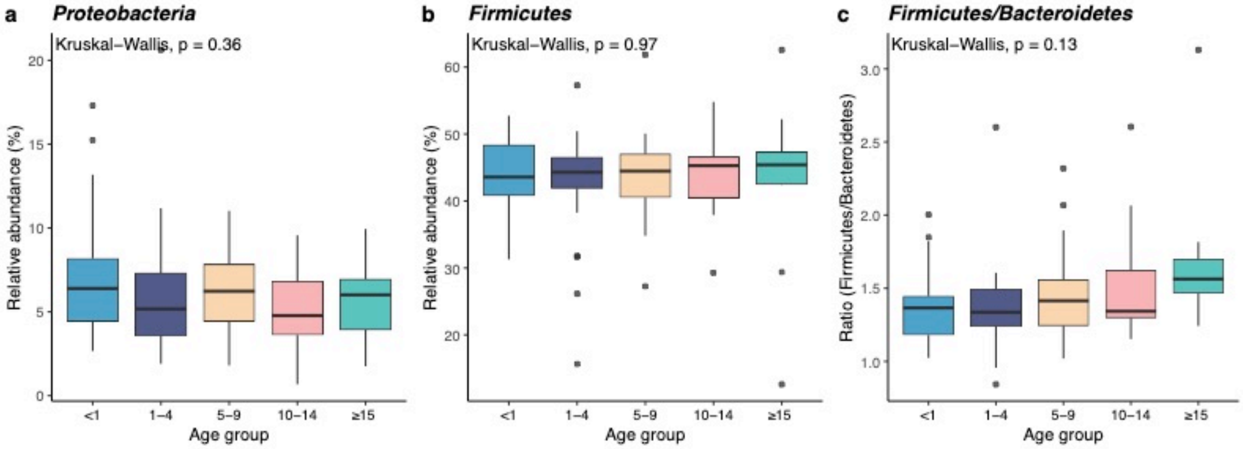

Figure S8. Relative abundances of the phyla (a) *Proteobacteria* and (b) *Firmicutes*, and the (c) ratio of *Firmicutes* to *Bacteroidetes* do not differ across age groups.

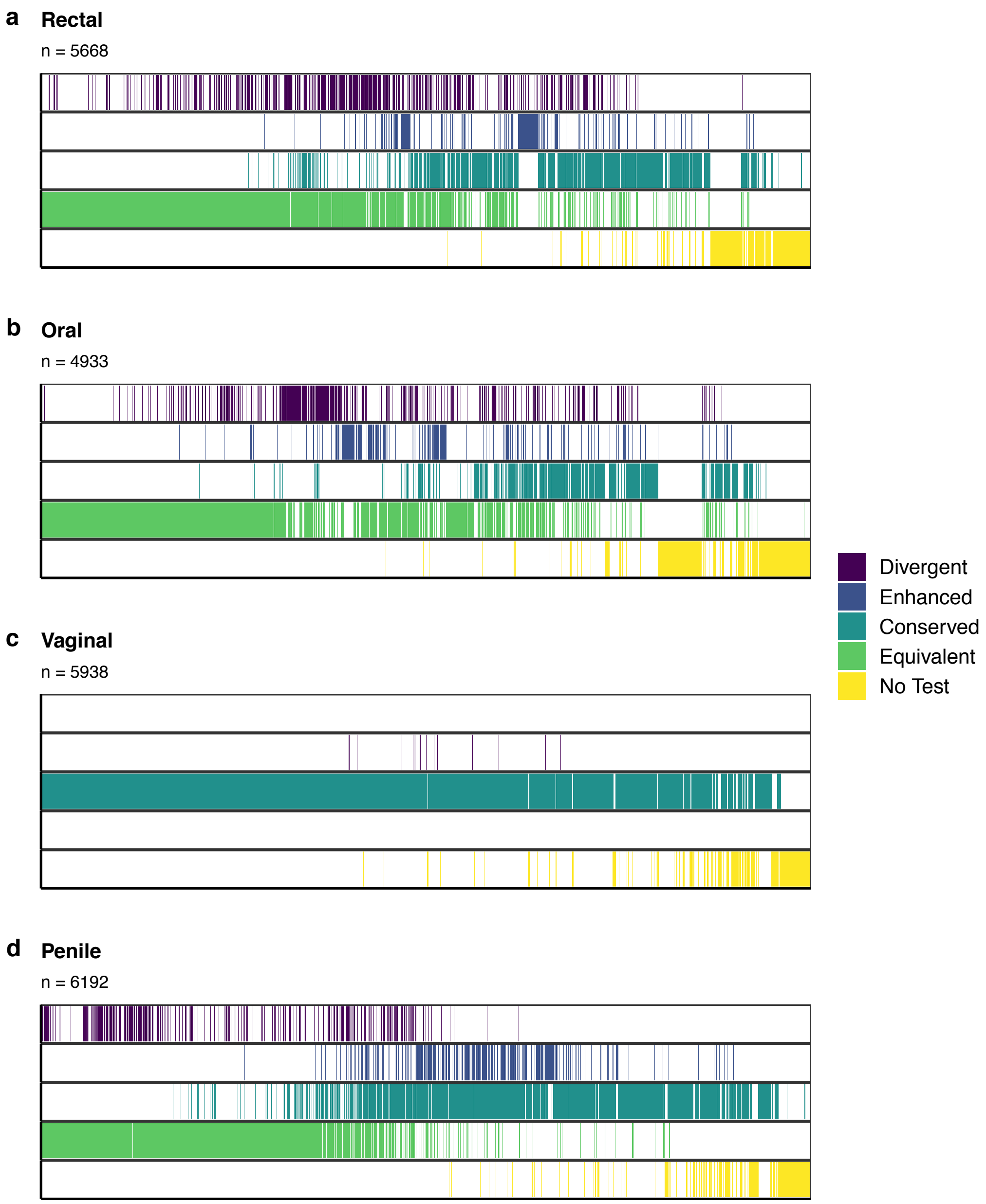

Figure S9. Differences in predicted microbial functions across age groups in (a) rectal, (b) oral, (c) vaginal, and (d) penile communities. Each vertical line is a predicted KEGG gene/function, classified by the CatFun function in FunkyTax as enhanced (frequency of function differs among groups but contributing community does not), divergent (frequency of function and contributing community differs among groups), conserved (frequency of function and contributing community does not differ among groups), or equivalent (frequency of function does not differ among groups but contributing community differs).

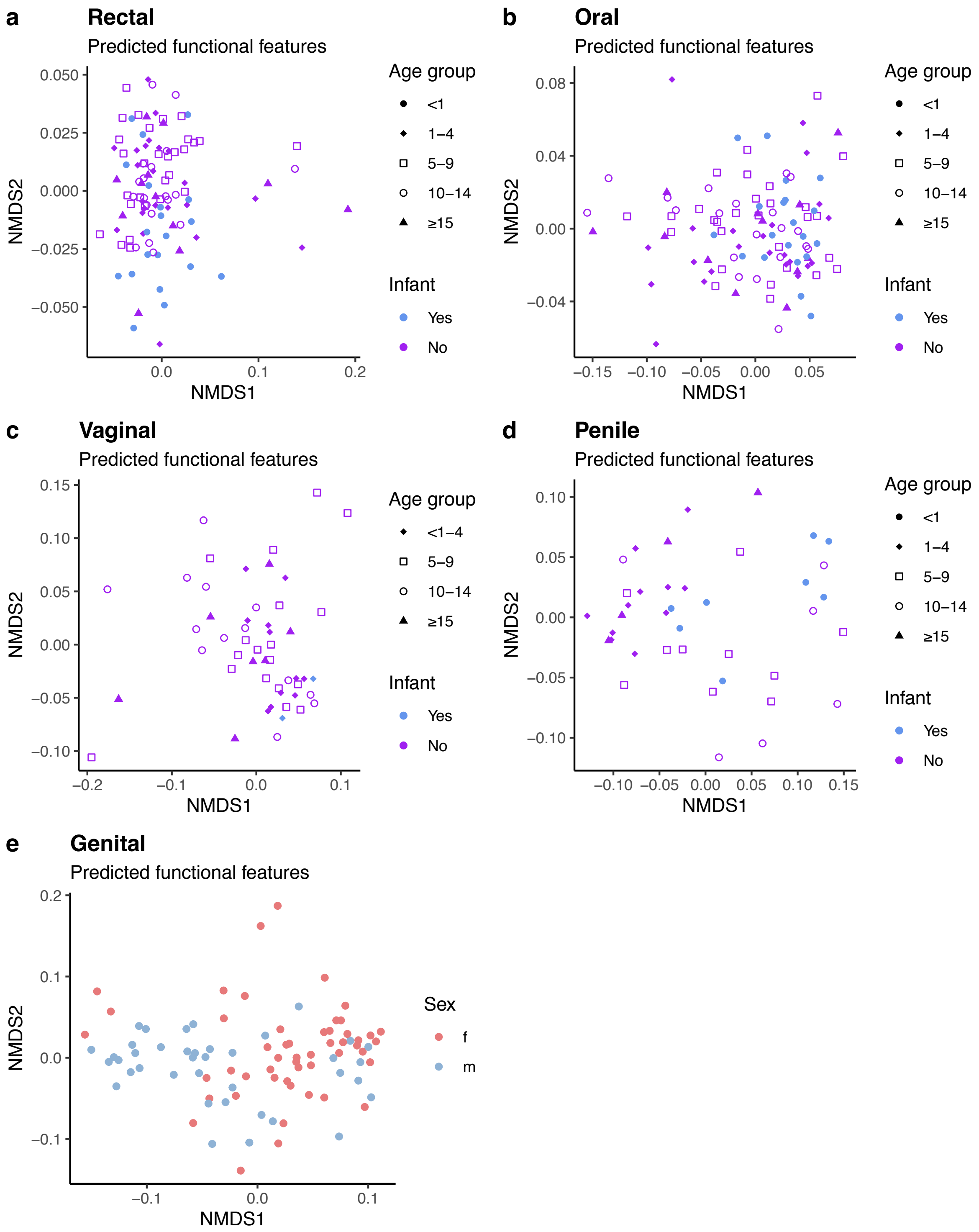

Figure S10. Bray-Curtis distances of predicted functional features of (a) rectal, (b) oral, (c) vaginal, (d) penile, and (e) genital microbial communities ordinated by NMDS.

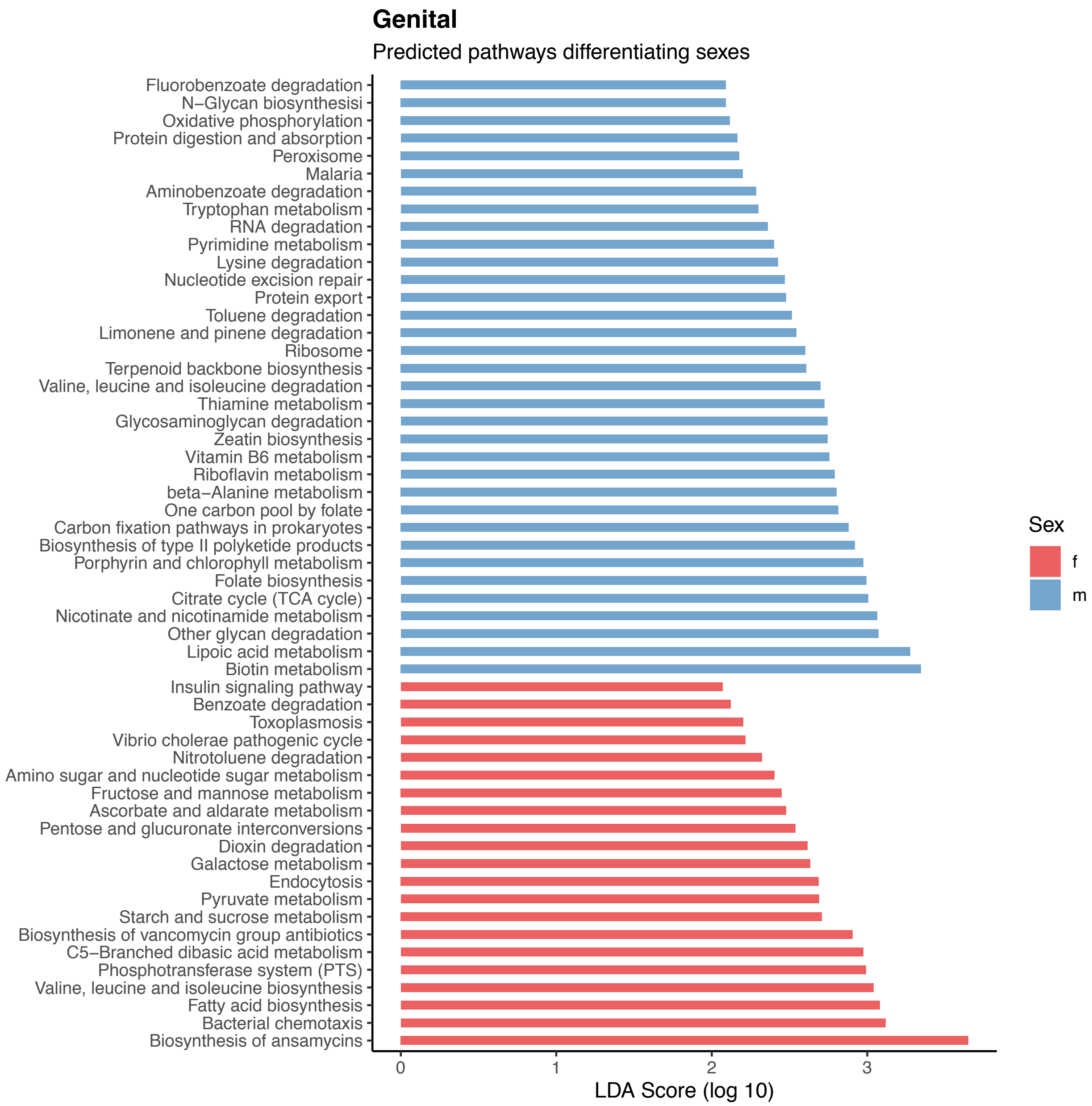

Figure S11. Functional pathways predicted by PiCrust2 that differentiate male and female genital communities. Pathways identified by LEfSe with LDA effect size ≥2 and alpha ≤ 0.01.
